# High-Dose Intravenous Vitamin C Combined with Docetaxel in Men with Metastatic Castration-Resistant Prostate Cancer: A Randomized Placebo-Controlled Phase II Trial

**DOI:** 10.1158/2767-9764.CRC-24-0225

**Published:** 2024-08-20

**Authors:** Channing J. Paller, Marianna L. Zahurak, Adel Mandl, Nicole A. Metri, Aliya Lalji, Elisabeth Heath, William K. Kelly, Christopher Hoimes, Pedro Barata, Jason Taksey, Dominique A. Garrison, Kartick Patra, Ginger L. Milne, Nicole M. Anders, Julie M. Nauroth, Jennifer N. Durham, Catherine H. Marshall, Mark C. Markowski, Mario A. Eisenberger, Emmanuel S. Antonarakis, Michael A. Carducci, Samuel R. Denmeade, Mark Levine

**Affiliations:** 1 Department of Medicine, School of Medicine, Johns Hopkins University, Baltimore, Maryland.; 2 Division of Biostatistics and Bioinformatics, Johns Hopkins University, Baltimore, Maryland.; 3 Barbara Ann Karmanos Cancer Institute, Detroit, Michigan.; 4 Thomas Jefferson University, Philadelphia, Pennsylvania.; 5 Duke Cancer Institute, Durham, North Carolina.; 6 Case Western Reserve University/University Hospitals, Cleveland, Ohio.; 7 Maryland Oncology Hematology, US Oncology, Annapolis, Maryland.; 8 Molecular and Clinical Nutrition Section, Digestive Diseases Branch, National Institute of Diabetes and Digestive and Kidney Diseases, National Institutes of Health, Bethesda, Maryland.; 9 Division of Clinical Pharmacology, Department of Medicine, Vanderbilt University Medical Center, Nashville, Tennessee.; 10 University of Minnesota Masonic Cancer Center, Minneapolis, Minnesota.

## Abstract

High-dose intravenous vitamin C (HDIVC) administered to produce pharmacologic concentrations shows promise in preclinical models and small clinical trials, but larger prospective randomized trials are lacking. We evaluated the clinical benefit of combining HDIVC with docetaxel in patients with progressive metastatic castration-resistant prostate cancer (mCRPC). In this double-blind, placebo-controlled phase II trial, 47 patients were randomized 2:1 to receive docetaxel (75 mg/m^2^ i.v.) with either HDIVC (1 g/kg) or placebo. Coprimary endpoints were PSA50 response and adverse event rates. Secondary endpoints included overall survival, radiographic progression-free survival, and quality of life measured using the Functional Assessment of Cancer Therapy-Prostate instrument. Correlative analyses included pharmacokinetics and oxidative stress markers. Eighty-nine percent of patients previously had three or more lines of therapy. The PSA50 response rate was 41% in the HDIVC group and 33% in the placebo group (*P* = 0.44), with comparable adverse event rates in both groups. There were no significant differences in Functional Assessment of Cancer Therapy-Prostate scores. The median radiographic progression-free survival was not significantly different between the HDIVC and placebo groups, with durations of 10.1 and 10.0 months (HR, 1.35; 95% confidence interval, 0.66–2.75; *P* = 0.40), respectively. The median overall survival was 15.2 months in the HDIVC group and 29.5 months in the placebo group (HR, 1.98; 95% confidence interval, 0.85–4.58; *P* = 0.11). HDIVC did not decrease F2-isoprostanes, indicators of oxidative stress. The study was suspended after prespecified interim analysis indicated futility in achieving primary endpoints. In this patient population, combining HDIVC with docetaxel did not improve PSA response, toxicity, or other clinical outcomes compared with docetaxel alone. Findings do not support the routine use of HDIVC in mCRPC treatment outside of clinical trials.

**Significance:**

This is the first randomized, placebo-controlled, double-blind trial to evaluate HDIVC in cancer treatment. The addition of HDIVC to docetaxel in patients with mCRPC does not improve PSA response, toxicity, or other clinical outcomes compared with docetaxel alone. The routine use of HDIVC in mCRPC treatment is not supported outside of clinical trials.

## Introduction

Vitamin C (ascorbic acid or ascorbate) is essential for humans. When taken orally, its concentrations in both plasma and tissues are tightly regulated by multiple mechanisms: intestinal absorption, tissue transport, renal reabsorption/excretion, and tissue utilization (1–4). However, tight physiologic control is bypassed when vitamin C is administered parenterally. In animals and humans, parenteral administration uniquely results in pharmacologic ascorbic acid concentrations, which persists until renal excretion restores homeostasis (4). Only pharmacologic ascorbic acid concentrations, not physiologic concentrations, produce extracellular hydrogen peroxide as a prodrug for reactive oxygen species, which is effective *in vitro* and in animal models in limiting the growth of a wide variety of cancers (5, 6).

Intravenous vitamin C (IVC) has a strong safety record, with only minimal adverse events (AE) reported among the approximately 10,000 individuals who undergo IVC treatment annually, including lethargy, fatigue, nausea, and vomiting in less than 1% (7). In small phase I/II clinical trials, IVC has shown promising efficacy across various cancers, including ovarian cancer, pancreatic cancer, glioblastoma, and multiple myeloma (8–15). Notably, trials that combined high-dose IVC (HDIVC) with chemotherapy for advanced cancers indicated improved quality of life and reduced toxicities (11, 16, 17). Specifically, one randomized controlled trial involving newly diagnosed patients with ovarian cancer revealed that the addition of IVC to first-line treatment with paclitaxel and carboplatin chemotherapy prolonged time to disease progression and suggested favorable trends in overall survival (OS). Additionally, ascorbate addition led to a marked reduction in low-grade toxicities associated with chemotherapy (11).

Metastatic castration-resistant prostate cancer (mCRPC) treatment poses a significant challenge, particularly when patients exhaust standard therapeutic avenues of androgen deprivation therapy and androgen receptor signaling inhibitors. Subsequent-line treatment options include docetaxel or cabazitaxel and are aimed at extending survival (18). The utility of taxane therapies is frequently limited by associated toxicities that encompass a spectrum of AEs ranging from low-grade symptoms like fatigue, nausea/vomiting, neuropathy, bone pain, and anorexia to more severe grade 3 to 4 AEs like neutropenia, anemia, and thrombocytopenia. Such treatment-related complications often necessitate infusion delays, dose adjustments, or even discontinuation of therapy. Approximately 11% to 35% of men with mCRPC receiving docetaxel experience dose interruptions, underscoring the need for alternative or adjunctive treatments (18, 19).

Because parenteral administration of pharmacologic doses of vitamin C inhibited growth of CRPC in an animal model (20), a noncomparative phase II clinical study was previously conducted, wherein a cohort of 20 men diagnosed with mCRPC received a regimen of single-agent IVC once weekly with step-up dosing to a target dose of 60 g over 12 weeks (0.74 g/kg; ref. 21). There were no reductions in PSA levels, oxidative damage markers (such as 8-oxoguanidine excretion), or improvements of bone metastases. However, the lack of response could be attributed to suboptimal dosing and single-agent therapy (21).

At present, there are no appropriately powered, randomized, prospective trials using HDIVC for the treatment of any cancer. We present here results from a randomized, placebo-controlled phase II clinical trial that evaluated the therapeutic potential of combining HDIVC with docetaxel in patients with mCRPC. We hypothesized that HDIVC would increase PSA response and/or mitigate toxicities.

## Materials and Methods

### Study design and participants

This randomized (2 HDIVC/docetaxel : 1 placebo/docetaxel), double-blind, placebo-controlled phase II trial (ClinicalTrials.gov identifier: NCT02516670) was approved by the Johns Hopkins University Institutional Review Board and conducted across six sites in the United States (Supplementary Table S1). Eligible patients were men ≥18 years with mCRPC who progressed as per Prostate Cancer Clinical Trials Working Group 3 (PCWG3) criteria (22). Patients had symptomatic disease or visceral metastases or qualified for docetaxel treatment due to disease progression despite androgen receptor signaling inhibitor therapy, and they had an Eastern Cooperative Oncology Group performance status (23) grade 0 or 1. Enrollment criteria excluded patients who had previously undergone chemotherapy for mCRPC but allowed patients who had received chemotherapy in the hormone-sensitive state. All study participants provided written informed consent approved by the Institutional Review Board. Full eligibility criteria are provided in the trial protocol (Supplementary Material Trial Protocol).

### Study procedures

Patients received docetaxel 75 mg/m^2^ i.v. every 3 weeks (day 1 of each cycle). The combination treatment group received HDIVC of 1 g/kg twice per week (first dose on day 1 of each cycle), whereas the control group received matching normal saline following the same schedule. Treatment continued until disease progression, unacceptable toxicities, or a maximum of eight cycles, followed by an optional open-label extension phase for all participants, in which patients received the combination of docetaxel and HDIVC until disease progression or toxicity. CT or MRI and bone scanning were performed at baseline, every 12 weeks, and 30 days after the last dose of the study drug. Blood tests and safety assessments were performed weekly and at the 30-day follow-up. The Functional Assessment of Cancer Therapy-Prostate (FACT-P) questionnaire was completed at baseline and on day 1 of cycles 4, 6, and 8. For F2-isoprostanes (F_2_-IsoP), blood was collected at baseline, immediately at the end of the infusion of HDIVC, or 60 minutes following the infusion. Further details are provided in the trial protocol (Supplementary Material Trial Protocol).

### Outcomes

Coprimary endpoints were the PSA50 response rate (≥50% decline in the PSA level from baseline at any time during the 24 weeks of treatment, PCWG2) and toxicity, defined as the worst grade of four AEs of interest (fatigue, nausea, bone pain, and anorexia) over the first 24 weeks of treatment. Secondary endpoints included radiographic progression-free survival (rPFS), defined as the time from randomization until soft-tissue lesion progression on CT or MRI (by RECIST version 1.1), bone lesion progression on bone scanning (by PCWG2 criteria), or death, whichever occurred first; OS; any AEs; and quality of life measured by the FACT-P scale. In a subsequent *post hoc* analysis, PSA responses were assessed, adjusting for prior exposure to docetaxel. The duration of PSA response was quantified from the first day of the response-acquiring cycle to the date of PSA progression—designated as the initial PSA increase equivalent to or exceeding 25% and 2 ng/mL above the nadir. Exploratory endpoints encompassed pharmacokinetics (PK) of plasma vitamin C and docetaxel and the pharmacodynamic measurement of plasma F_2_-IsoPs. Detailed endpoint definitions are provided in the trial protocol (Supplementary Material Trial Protocol).

### Statistical analysis

The sample size of 63 patients (42 in the HDIVC treatment group and 21 in the control group) would provide 80% power to detect the hypothesized 35% absolute improvement in PSA response with a one-sided 5% Fisher exact test. Efficacy and safety analyses were conducted on a modified intention-to-treat basis, incorporating all treated patients. To preserve the coprimary significance level at 15%, the α-level was set at 5% for PSA response and 10% for toxicity. Detailed description of the statistical analysis methods is provided in the Supplementary Methods.

### Toxicity

Grading of the coprimary toxicity outcome was categorical (no toxicities, grades 1–2, and grades 3–4), and the Cochran–Armitage trend test was used for analysis. Separate queries were conducted to analyze AE data specific to each study drug. The earliest instance of each AE with the highest grade and attribution combination for each patient and AE type was extracted. It is important to note that some patients experienced multiple types of AEs and recurring episodes of the same Common Terminology Criteria for Adverse Events code. In such cases, the earliest instance of that AE with the highest grade and attribution combination was reported.

### rPFS and OS

The median, 12-month, and 24-month rPFS and OS were estimated with the Kaplan–Meier (KM) method, and HRs and 95% confidence intervals (CI) with Cox proportional-hazards regression models. The median follow-up was calculated using the KM method.

### PSA response rate

The Fisher exact test was used to compare PSA response rates. An interim analysis of PSA response applied a predictive probability approach; the trial would halt for futility if, after 30 patients completed the required PSA follow-up, the probability of concluding the trial with significant results was below 5%. *Post hoc* analysis involved the exact Cochran–Mantel–Haenszel test to explore the correlation of HDIVC treatment with PSA response, factoring in prior docetaxel exposure. An assumption of the Cochran–Mantel–Haenszel stratified analysis is that the ORs within each stratum (prior docetaxel, yes or no) are homogeneous. The Breslow–Day test for homogeneity of ORs is used to confirm this assumption for a stratified analysis. For the duration of PSA response, the end dates for several responders are censored, requiring a KM analysis for comparison. Statistical analyses were performed using R software, version 4.1.3 (Comprehensive R Archive Network, www.cran.r-project.org), with a *P* value <0.05 defining statistical significance.

### FACT-P

The FACT-P comprises two main components: FACT-General, a 27-item self-report questionnaire with four subscale domains (physical, social/family, emotional, and functional well-being) designed to measure general quality of life in patients with cancer, and a 12-item prostate cancer subscale (PCS) tailored to assess prostate cancer–specific quality of life. The FACT-P total score is calculated by summing the scores from the FACT-General subscales and the PCS, with higher total scores indicating better quality of life. Two additional scores from the FACT-P questionnaire were used: the FACT Advanced Prostate Symptom Index score, which includes eight items from the FACT-P, and the FACT-P PCS pain-related score, which comprises four questions from the FACT-P specifically addressing pain. As with the overall FACT-P, higher scores on these indices reflect better health-related quality of life. FACT-P total scores at cycles 4, 6, and 8 were evaluated with analysis of covariance, adjusting for baseline FACT-P total scores.

### PK

PK parameters (maximum concentration and AUC) were compared between treatment arms using the Wilcoxon rank-sum test, with data presented as the mean ± SD. A *P* value <0.05 was defined to be statistically significant. Standard noncompartmental methods in Phoenix WinNonlin version 8.3 (Certara) were used to calculate the PK parameters from individual concentration–time data. Statistical analysis was conducted using JMP Statistical Discovery software version 7.0 (SAS Institute Inc.). Ascorbic acid levels were measured by high-performance liquid chromatography with coulometric electrochemical detection.

### F2-IsoPs

Concentrations of F_2_-IsoPs were determined at Vanderbilt Eicosanoid Core Laboratory using gas chromatography/negative ion chemical ionization mass spectrometry assays. Whole blood was centrifuged at 4,000 *g* for 10 minutes to yield plasma, and 0.5 to 1 mL of plasma was used for the quantification of F_2_-IsoPs. The sample was derivatized to the pentafluorobenzyl ester, trimethylsilyl ether derivative for gas chromatography/negative ion chemical ionization mass spectrometry analysis. The lower limit of sensitivity was about 5 pg. The precision of the assay was ±6%, and the accuracy was 96%. The final results were standardized and expressed as nanograms per milligram of creatinine. Comparisons were made between study arms based on infusions at cycles 4 and 6 using the two-sample *t* test.

### Data availability

The data generated in this study are available upon request from the corresponding author. Data are not publicly available to maintain the protection of patient privacy.

## Results

### Patients

**Figure 1:**
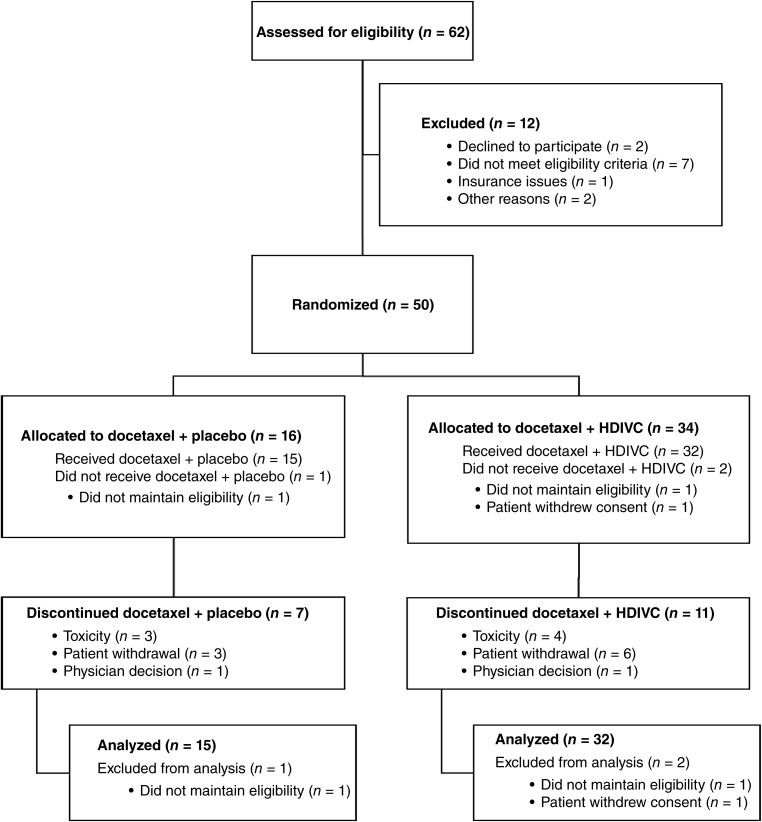
CONSORT diagram. The diagram shows eligibility assessment, randomization, allocation, discontinuations, and numbers of subjects analyzed.

Between June 20, 2016, and September 21, 2021, 62 patients were screened, and 50 were randomized in a 2:1 ratio, with 34 in the HDIVC group and 16 in the control group (Fig. 1). Baseline demographic and clinical characteristics (Table 1) were balanced between groups. The mean age was 74 years (SD, 7.6); 72% of patients were white, and 60% had an Eastern Cooperative Oncology Group performance status grade of 1. The median baseline PSA level was 108.3 ng/mL (range 0.3–2,102.3 ng/mL). Of the 50 randomized patients, 47 received their assigned treatments (32 in the combination group and 15 in the control group). Three patients were not treated because of withdrawal, elevated creatinine, or low hemoglobin. Patients in the HDIVC arm had a median of seven (IQR, 4–8.25) cycles of docetaxel, whereas the control group had a median of eight cycles (IQR, 1.5–8.5). Twenty-four patients completed at least eight cycles of study treatment (16 in the HDIVC group and 8 in the control group, with 9 and 4 moving on to the extension phase, respectively). The most common reasons for treatment discontinuation were disease progression (38%) and patient withdrawal (19%). The median follow-up was 27 months (range, 3.8–45 months) in the HDIVC treatment group and 32.6 months (range, 1.5–37.8) in the control group.

**Table 1:** Demographic and baseline characteristics

Characteristic	Total treated (*n* = 47)	Docetaxel alone (*n* = 15)	Docetaxel + HDIVC (*n* = 32)
Gender, male [*n*, (%)]	47 (100%)	15 (100%)	32 (100%)
Age, years (mean, SD)	74 (7.6)	73 (9.4)	74 (6.8)
Race, *n* (%)			
Asian	1 (2%)	0 (0%)	1 (3%)
Black	11 (23%)	6 (40%)	5 (16%)
White/Caucasian	34 (72%)	9 (60%)	25 (78%)
Unknown	1 (2%)	0 (0%)	1 (3%)
Gleason grade group, *n* (%)			
Group 1	3 (6%)	1 (7%)	2 (6%)
Group 2	2 (4%)	0 (0%)	2 (6%)
Group 3	2 (4%)	0 (0%)	2 (6%)
Group 4	14 (30%)	3 (20%)	11 (34%)
Group 5	19 (40%)	8 (53%)	11 (34%)
Not evaluable	7 (15%)	3 (20%)	4 (12%)
ECOG performance status grade, *n* (%)			
0	18 (40%)	6 (40%)	12 (40%)
1	27 (60%)	9 (60%)	18 (60%)
PSA at baseline, median (IQR)	108.3 (2.1–2102.3)	107.0 (2.1–999.0)	123.7 (0.3–2102.3)
Sites of metastasis at baseline, *n* (%)			
Bone	45 (96%)	13 (87%)	32 (100%)
Visceral	9 (19%)	3 (20%)	6 (19%)
Lymph nodes	3 (9%)	0 (0%)	3 (9%)

Prior therapies, *n* (%)			
Radiotherapy	25 (53%)	10 (67%)	15 (47%)
Prostatectomy	15 (32%)	5 (33%)	10 (31%)
Palliative radiation	8 (17%)	3 (20%)	5 (16%)
Lines of therapy, *n* (%)			
1	1 (2%)	0 (0%)	1 (3%)
2	4 (9%)	0 (0%)	4 (12%)
3	15 (32%)	5 (33%)	10 (31%)
≥4	27 (57%)	10 (67%)	17 (53%)
Systemic therapy, *n* (%)			
ADT^a^	44 (94%)	14 (93%)	30 (94%)
ARSI^b^	46 (98%)	15 (100%)	31 (97%)
Chemotherapy^c^	10 (21%)	5 (33%)	5 (16%)
Other systemic therapies^d^	37 (79%)	13 (87%)	24 (75%)

Abbreviations: ADT, androgen deprivation therapy; ARSI, androgen receptor signaling inhibitor; ECOG, Eastern Cooperative Oncology Group.

a ADT, leuprolide/unspecified ADT.

b ARSI, bicalutamide/apalutamide/darolutamide/enzalutamide/abiraterone/nilutamide.

c Chemotherapy, docetaxel/cabazitaxel for hormone-sensitive metastatic prostate cancer.

d Other systemic therapies: LY2157299/galunisertib (4), GSK525762/molibresib (1), cabozantinib (1), Zenith 006 (1), ESK981 (1), BAT (1), Inovio vaccine trial (1), RESTORE (2), SIP-T (14), ketoconazole (2), OSI-906 dual inhibitor (1), anti-PDL1/pembrolizumab (2), ONC201 (1), TAK-700 (1), TRX518/GITR agonist (1), GS-5829/BET inhibitor (2), and double androgen blockade (1).

### Primary outcomes

In the HDIVC group, 41% of patients (13 of 32) achieved a PSA50 response compared with 33% (5 of 15) in the control group (Table 2). However, this difference failed to reach the anticipated 35% absolute improvement (*P* = 0.44). *Post hoc* analysis stratified by prior docetaxel use corroborated these findings (OR, 1.26; 95% CI, 0.29–5.91; Supplementary Table S2). Due to an insufficient PSA response rate in the interim analysis, accrual to the trial was suspended by the Data and Safety Monitoring Board for futility. Table 3 presents the worst grade of four AEs of interest (fatigue, nausea, bone pain, and anorexia) encountered by patients during the 24-week treatment. Overall, the groups exhibited comparable AE profiles (Supplementary Tables S3 and S4). Most reported AEs were of grades 1 to 2, comprising 69% in the HDIVC group and 60% in the control group. Grade 3 to 4 AEs emerged in 6% of patients in the HDIVC group, with none in the control (*P* for trend = 0.90). More patients in the HDIVC treatment group experienced grade 1 to 2 anorexia (treatment, 28%; control, 7%); conversely, more patients in the control group exhibited grade 1 to 2 fatigue (treatment, 25%; control, 33%) and bone pain (treatment, 6%; control, 13%). Grade 3 to 4 AEs of interest were only observed in the HDIVC treatment group, specifically fatigue (3%) and nausea (3%).

**Table 2:** PSA response rate

PSA50	Docetaxel + HDIVC (*n* = 32)	Docetaxel + placebo (*n* = 15)	Combined (*n* = 47)
No	59% (19)	67% (10)	62% (29)
Yes	41% (13)	33% (5)	38% (18)

**Table 3:** AEs for coprimary endpoints

	Docetaxel + HDIVC (*n* = 32)		Docetaxel + placebo (*n* = 15)	
Severity, *n* (%)	Grades 1–2: 22 (69%)	Grades 3–4: 2 (6%)	Grades 1–2: 9 (60%)	Grades 3–4: 0 (0%)
AE				
Anorexia	9	0	1	0
Bone pain	2	0	2	0
Fatigue	9	1	5	0
Nausea	2	1	1	0

### Secondary outcomes

**Figure 2:**
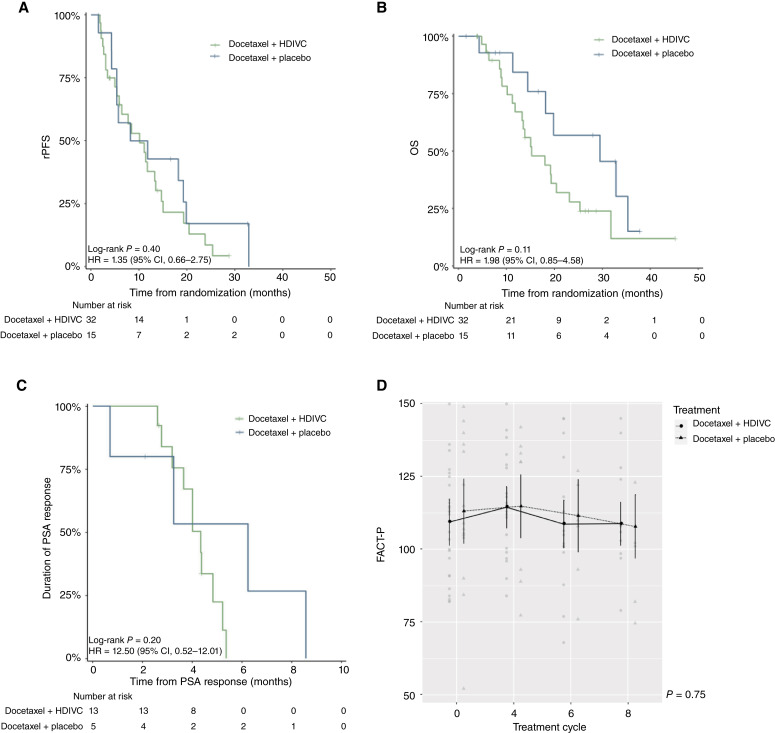
Key secondary endpoints. Key secondary endpoints include rPFS, OS, duration of PSA response, and quality of life measured using the FACT-P instrument. **A,** rPFS and (**B**) OS are presented for docetaxel + HDIVC vs. docetaxel + placebo. The KM method was used to estimate the median, 12-month, and 24-month rates of rPFS and OS. **C,** Duration of PSA response and (**D**) FACT-P scores are presented for docetaxel + HDIVC vs. docetaxel + placebo.

The median rPFS was 10.1 months (95% CI, 5.85–14.7) in the HDIVC group and 10.0 months (95% CI, 5.32–NA) in the control group (HR, 1.35; 95% CI, 0.66–2.75; *P* = 0.40; Fig. 2A). The HDIVC group demonstrated a nonsignificantly shorter median OS—15.2 months (95% CI, 13.2–25.3)—in comparison with 29.5 months (95% CI, 18.1–NA) in the control group (HR, 1.98; 95% CI, 0.85–4.58; *P* = 0.11; Fig. 2B).

In this study, 18 patients achieved a PSA response, with 5 in the control arm and 13 in the HDIVC arm. A *post hoc* analysis assessed the duration of PSA response, calculated from the initial day of the cycle wherein the response was first noted to the day of PSA progression defined as the initial PSA increase ≥25% and ≥2 ng/mL above the nadir. The median duration of PSA response (Fig. 2C) was shorter in the HDIVC group than in the control group, 4.3 versus 6.2 months, respectively (HR, 2.5; 95% CI, 0.52–12; *P* = 0.25).

The incidence of grade 1 to 2 AEs of any type was similar between the HDIVC (94.1%) and control groups (93.8%). However, the incidence of grade 3 to 4 AEs of any type was more prevalent in the HDIVC group (64.7%) compared with the control group (50%). The most common AEs of any grade in the HDIVC group compared with the control group were fatigue (72% vs. 56%), diarrhea (72% vs. 19%), alopecia (56% vs. 31%), and nausea (50% vs. 31%), respectively. Two patients in the HDIVC group (6%) experienced venous thromboembolic events—deep vein thrombosis and renal vein thrombosis—during the treatment period that were not related to HDIVC or docetaxel. Eleven patients reported twenty-seven serious AEs, none of which were related to HDIVC. Of those 27 serious AEs, 18 were unrelated, 6 were definitely related, 2 were possibly related, and 1 was unlikely related to docetaxel. The most frequently occurring serious AE was febrile neutropenia, definitely attributable to docetaxel in three of four patients (Supplementary Tables S5–S8). In the HDIVC group, 21% of patients had a dose reduction of docetaxel (75–60 mg/m^2^) and 2% had a dose reduction of HDIVC (1–0.75 g/kg), whereas in the control group, 2% of patients had a dose reduction of docetaxel.

At baseline, 89% of patients (42 of 47) completed the FACT-P questionnaire, with the median total scores of 113 (range, 82–150) in the treatment group and 112 (range, 52.2–149) in the control group. Compliance for subsequent treatment cycles, based on an expected number of patients that were alive and not having progressed, declined at cycle 4 [29/41 patients (71%)], cycle 6 [24/36 patients (67%)], and cycle 8 [16/29 patients (55%)] but remained above 50%. Comparisons of FACT-P total scores between arms on day 1 of cycles 4, 6, and 8 were not significant (Fig. 2D; Supplementary Fig. S1; Supplementary Tables S9–S12).

### PK results

PK data for docetaxel were collected from 17 patients, with 7 patients (7/15, 47%) in the cohort receiving docetaxel with placebo and 10 patients (10/32, 31%) receiving docetaxel with HDIVC. The analysis revealed minimal changes in the maximum concentration achieved for docetaxel alone (2,367.1 ± 730.7 ng/mL) compared with when combined with HDIVC (2,762.0 ± 787.6 ng/mL; *P* = 0.28). Additionally, there was no statistically significant difference in dose-normalized exposure for docetaxel alone (3,124.7 ± 404.0 ng × h/mL) or in combination with HDIVC (3,623.4 ± 1,432.9 ng × h/mL; *P* = 0.33). However, we observed a trend toward higher docetaxel exposure in the plasma of patients receiving the combination treatment.

PK data for vitamin C were voluntary and only available for five subjects who received docetaxel with HDIVC and two subjects who received docetaxel alone (Supplementary Fig. S2). Vitamin C concentrations were as expected in those who received HDIVC (5, 24) and in controls (1–4).

### Pharmacodynamic measure of F_2_-IsoPs

F_2_-IsoPs (15-F_2t_-IsoP, PGF_2α_, 5-F_2t_-IsoP, and 5-F_2c_-IsoP) are biomarkers for oxidative stress. We hypothesized that if decreases in AEs occurred with HDIVC treatment, there might be associated changes in F_2_-IsoPs in plasma. F_2_-IsoPs were analyzed at completion of vitamin C infusions and 60 minutes postinfusion, and they were compared with baseline concentrations at cycles 4 and 6. There were no reductions in F_2_-IsoP concentrations when compared with baseline measurements, although sample sizes were limited (Supplementary Tables S13–S17).

## Discussion

This is the first randomized, placebo-controlled, double-blind trial to evaluate HDIVC in cancer treatment. Here, we investigated PSA response rates and selected chemotherapy-related toxicities (fatigue, nausea, bone pain, and anorexia) in patients with mCRPC who received a combination of HDIVC with standard-of-care docetaxel. We also studied the impacts of HDIVC on other AEs, rPFS, OS, and quality-of-life metrics. Comparison between HDIVC and control groups revealed no significant difference in PSA response (PSA50), irrespective of prior docetaxel use in the hormone-sensitive setting. Similar patterns were observed across all endpoints without significant differences between groups. Interim analyses highlighting inadequate PSA responses led to trial suspension due to futility. Although the final sample size was limited by the futility analysis, the trial efficiently evaluated PSA50 response and toxicity outcomes in this patient population. The PSA50 response in the HDIVC arm at the time of the interim analysis, 41%, was below the trial design’s hypothesized null, 45%, which was based on the TAX 327 trial (18). Additionally, with the posterior probability of having a successful trial less than 5% and median rPFS and OS both being shorter in the HDIVC arm at the interim, the decision to halt the trial could be made with a savings of 20% of the original planned size (50 vs. 63). Compared with controls, in men who received docetaxel and HDIVC, there were nonsignificant trends of increased AEs, reduced OS, and higher docetaxel exposure in plasma.

Two factors that might explain the lack of efficacy are frequency of administration and dose of HDIVC. Daily administration is common in preclinical models (20). However, twice-weekly administration, used here and in other clinical trials (10, 11, 15), may have been insufficient for mCRPC and was logistically challenging. Because most HDIVC safety data are for doses at or below 1 g/kg, we were reluctant to increase dosing (6, 11, 13–15, 25).

Other factors may have contributed to the lack of efficacy. The trial size was limited, and 89% of those enrolled had previously failed three or more lines of prior therapy. Prespecified coprimary endpoints may have been overly optimistic, such that the trial, in retrospect, perhaps was underpowered. The trend toward higher docetaxel exposure in the combination arm might have contributed to observed toxicity. We also noted that controls in this study lived unexpectedly longer (29.5 months) than historical controls from previous trials of docetaxel in combination with other treatments (range, 17.6–22 months; refs. 26–30).

Investigating alternative combination strategies with parenteral ascorbate may present solutions to existing clinical challenges. A recent preclinical study (bioRxiv 2023.03.23.533944) explored the synergistic potential of combining pharmacologic vitamin C concentrations with three distinct PARP inhibitors (niraparib, olaparib, and talazoparib) utilizing models of CRPC. Combination treatment was synergistic, leading to a significant delay in tumor growth compared with the groups treated with monotherapy (bioRxiv 2023.03.23.533944). Combination therapy of parenteral ascorbate may also be synergistic with checkpoint inhibitors and chemotherapy in animal and cell models (24, 31, 32). Certain patient subgroups with specific genetic mutations may be particularly susceptible to combination therapies involving HDIVC. For example, emerging preclinical studies indicate that HDIVC could display increased effectiveness in cancers with mutations in *KRAS*, *BRAF*, *TET2*, *IDH1*, *IDH2*, *VHL*, *FH*, or *SDH* and in those with mismatch repair deficiencies or high expressions of GLUT1, and at least some of these gene mutations are associated with prostate cancer (32–36).

Preclinical data support vitamin C efficacy in prostate cancer primarily through a mechanism involving generation of hydrogen peroxide in extracellular fluid by pharmacologic concentrations of vitamin C. Although nearly 80% of tested cancer cells show responsiveness to such levels of vitamin C, approximately 20% do not (24). Nonresponsiveness in some cases can be attributed to increased production of the enzyme catalase, which dismutates (detoxifies) hydrogen peroxide (37). Catalase is upregulated in prostate cancer (38), serving to counteract the effects of hydrogen peroxide–derived oxidants (5, 10, 14, 25, 39). Such upregulation would likely seem as resistance to pharmacologic ascorbic acid, potentially explaining the lack of efficacy observed in our heavily pretreated population. Recent preclinical data corroborate these findings and hint at the potential treatment utility of inhibiting catalase in mCRPC (40).

HDIVC showed no efficacy and a nonsignificant trend toward harm when used in combination with docetaxel for the treatment of men with mCRPC. Additional studies should focus on identifying better dosing strategies for HDIVC and appropriate drugs to be used in combination. Lack of efficacy seen in this trial should not be interpreted as justification to abandon other clinical trials of pharmacologic ascorbate. In addition to explanations above, clinical trials for other agents showed that different cancer types in humans required trials for several different cancers before efficacy was shown. Despite current lack of appropriately powered randomized trials for HDIVC, it is difficult to reconcile published clinical data with overall futility (6, 8, 11, 13–15, 25). The mechanism of pharmacologic ascorbate action as a prodrug to generate reactive oxygen species is predictive of efficacy in many tumor types with minimal toxicity (5, 6, 24), and encouraging clinical results were found when ascorbate was included as primary therapy, in contrast to this trial (11, 14, 25, 41, 42). Clinicians should await results of ongoing prospective trials and initiation of others in which HDIVC is added to standard-of-care modalities in treatment-naïve subjects.

## Supplementary Material

Supplementary Methods Supplementary methods include study design and participants, outcomes and statistical analysis, FACT-P questionnaire, pharmacokinetics, and F2-Isoprostanes

Table S1 Table S1 shows Participating Sites and the Number of Patients Enrolled in the Trial at Each Institution

Table S2 Table S2 shows Association of HDIVC Treatment with PSA Response Adjusting for Prior Docetaxel Exposure

Table S3 Table S3 shows Adverse Events Included in the Co-Primary Endpoint: All Occurrences

Table S4 Table S4 shows Adverse Events Included in the Co-primary Endpoint: Worst Grade by Patient

Table S5 Table S5 shows AEs with Attribution Possible, Probable or Definite to Treatment (HDVIC or Placebo) listed by types and grades

Table S6 Table S6 shows AEs with Attribution Possible, Probable or Definite to Docetaxel listed by types and grades

Table S7 Table S7 shows Serious Adverse Events (N = 11 patients), by Type and Grade. Attributions Assigned Docetaxel versus HDIVC or Placebo

Table S8 Table S8 shows Prevalence of adverse events

Table S9 Table S9 shows FACT-P Total Scores: Baseline, Cycles 4, 6, and 8

Table S10 Table S10 shows ANCOVA Least-squares Means for Cycle 4, 6, and 8 FACT-P Total by Study Arm

Table S11 Table S11 shows FACT-P Change (on Study Minus Baseline) Scores

Table S12 Table S12 shows Comparison of FACT-P Change Scores Between Study Arms: Mean Difference Docetaxel + HDVIC Minus Docetaxel + Placebo

Table S13 Table S13 shows Comparison of F2-Isoprostanes at Baseline between Treatment and Control Arms

Table S14 Table S14 shows Comparison of F2-Isoprostanes Control and Intervention Changes (post - pre) Immediately after Cycle 4

Table S15 Table S15 shows Comparison of F2-Isoprostanes Control and Intervention Changes (post - pre) Immediately after Cycle 6

Table S16 Table S16 shows Comparison of F2-Isoprostanes Control and Intervention Changes 60 minutes after Cycle 4

Table S17 Table S17 shows Comparison of F2-Isoprostanes Control and Intervention Changes 60 minutes after Cycle 6

Figure S1 Figure S1 shows FACT-P scores

Figure S2 Figure S2 shows Plasma ascorbic acid concentrations

Supplementary: Trial Protocol Trial Protocol shows the details of the study
