# Supplementary material for: High-Dose Intravenous Vitamin C Combined with Docetaxel in Men with Metastatic Castration-Resistant Prostate Cancer: A Randomized Placebo-Controlled Phase II Trial
Source: Cancer Res Commun. 2024 Aug 20;4(8):2174–82. doi: 10.1158/2767-9764.CRC-24-0225 (PMC11333993; doi:10.1158/2767-9764.CRC-24-0225)
Supplement: Supplementary Methods — include study design and participants, outcomes and statistical analysis, FACT-P questionnaire, pharmacokinetics, and F2-Isoprostanes [file crc-24-0225_supplementary_methods_suppsm.docx]

**SUPPLEMENTARY APPENDIX**

Table of Contents

[SUPPLEMENTARY METHODS 2](#_Toc172031240)

[Study Design and Participants 2](#_Toc172031241)

[Outcomes and Statistical Analysis 2](#_Toc172031242)

[FACT-P Questionnaire 2](#_Toc172031243)

[Pharmacokinetics 3](#_Toc172031244)

[F2-Isoprostanes 3](#_Toc172031245)

[REFERENCES 4](#_Toc172031246)

# SUPPLEMENTARY METHODS

## Study Design and Participants

The study was approved by the Johns Hopkins University (JHU) Institutional Review Board (IRB) and conducted across six sites in the United States, including Johns Hopkins University School of Medicine Sidney Kimmel Comprehensive Cancer Center, Sibley Memorial Hospital, Anne Arundel HSRI, Barbara Ann Karmanos Cancer Institute, University Hospitals Seidman Cancer Center, and Thomas Jefferson University Hospital. The study was activated on June 20, 2016, and closed on September 21, 2021.

## Outcomes and Statistical Analysis

The safety analysis set includes all patients who received the study treatment at least once during the trial. Within the safety analysis, comparisons are made between the different study arms. The intent-to-treat (ITT) analysis set includes all randomized patients. The full analyses set (FAS), or modified ITT (mITT), is derived from the ITT set by excluding three patients who did not receive any treatment due to specific circumstances: two of these patients withdrew from the study early, while the third patient was unable to start treatment due to low hemoglobin levels.

The co-primary trial design hypotheses, based on the TAX 327 trial(1), were that PSA response could be improved from 45% in control to 80% in the experimental group and that there would be a trend of decreasing grade AEs of four types: fatigue, nausea, bone pain, and anorexia. The sample size of 63 patients (42 in the HDIVC treatment group, 21 in the control group) would provide 80% power to detect the hypothesized 35% absolute improvement in PSA response with a one-sided 5% Fisher’s exact test. To preserve the co-primary significance level at 15%, the significance level was set at 5% for PSA response and 10% for toxicity.

The Cochran-Mantel-Haenszel (CMH) test assesses the association of HDIVC treatment with PSA response, adjusting for prior docetaxel exposure. An assumption of the Cochran-Mantel-Haenszel stratified analysis is that the odds ratios within each stratum (prior docetaxel, yes or no) are homogeneous. The Breslow-Day test for homogeneity of odds ratios is used to confirm this assumption for a stratified analysis.

For the co-primary toxicity outcome, we assessed the most severe grade experienced by each patient among four specific adverse events (fatigue, nausea, bone pain, and anorexia) over the 24-week treatment period. We relied on simulated clinical trial data to estimate the required sample size for the toxicity endpoint. In these simulations, we employed the Cochran-Armitage test to compare observed proportions in the two treatment groups, categorized by toxicity grade (i.e., grade 0, grades 1-2, and 3-4). We assumed that, on average, 16% of patients in the control group would experience a grade 3-4 toxicity related to one of the four specified adverse events. Furthermore, we anticipated a decreased proportion of these events in the docetaxel + HDIVC treatment arm across the three categories: grade 0, grade 1-2, and 3-4. We conducted separate queries to analyze adverse event data specific to each study drug. We extracted the earliest instance of each adverse event with the highest grade and attribution combination for each patient and AE type. It is important to note that some patients experienced multiple types of adverse events and recurring episodes of the same CTCAE code. In such cases, we reported the earliest instance of that adverse event with the highest grade and attribution combination.

The interim monitoring for this study included a futility analysis for PSA response halfway through the study and continuous monitoring of only those safety events potentially related to the infusion of HDIVC. The study design did not include an interim analysis of the co-primary toxicity endpoint. Safety monitoring events that, if observed, would likely be attributable to the addition of HDIVC were sclerosis, preventing infusion, and kidney stones requiring cessation of treatment.

## FACT-P Questionnaire

We used the FACT-P questionnaire, a well-established tool for evaluating our participants' quality of life (QoL). The FACT-P comprises two main components: FACT-General, a 27-item self-report questionnaire with four subscale domains (physical, social/family, emotional, and functional well-being) designed to measure general QoL in cancer patients, and a 12-item prostate cancer subscale (PCS) tailored to assess prostate cancer-specific QoL. The FACT-P total score is calculated by summing the scores from the FACT-General subscales and the PCS, with higher total scores indicating better QoL. Clinically significant changes in FACT-P total scores referred to as the minimally important difference (MID) range, have been reported as falling between 6 to 10 points.(2)

Two additional scores from the FACT-P questionnaire were used: the FACT Advanced Prostate Symptom Index (FAPSI) score, which includes eight items from the FACT–P, and the FACT-P PCS pain-related score, which comprises four questions from the FACT-P specifically addressing pain. As with the overall FACT-P, higher scores on these indices reflect better Health-Related Quality of Life (HRQoL).

## Pharmacokinetics

Pharmacokinetic parameters (Cmax and AUC) were compared between treatment arms using the Wilcoxon Rank-Sums test. Data for Cmax and AUC is presented as arithmetic mean values plus or minus standard deviation. A P-value <0.05 was defined to be statistically significant. Standard noncompartmental methods in Phoenix WinNonLin version 8.3 (Certara, St Louis, MO, USA) were used to calculate the pharmacokinetic parameters from individual concentration-time data. Statistical analysis was conducted using JMP® Statistical Discovery software version 7.0 (SAS Institute Inc., Cary, NC, USA). Ascorbic acid levels were measured by HPLC with coulometric electrochemical detection (3,4)

## F2-Isoprostanes

We collected blood at baseline, immediately at the end of the infusion of HDIVC, or sixty minutes following the infusion. Concentrations of F2-isoprostanes were determined at Vanderbilt Eicosanoid Core Laboratory using gas chromatography/negative ion chemical ionization mass spectrometry (GC/NICI MS) assays, as previously described in detail.(5) Briefly, whole blood was centrifuged at 4000g for 10 min to yield plasma. 0.5–1 mL of plasma was used for the quantification of F2-isoprostanes. The sample was derivatized to the pentafluorobenzyl ester, tri- methylsilyl ether derivative for GC/NICI-MS analysis. The lower limit of sensitivity was about 5 pg. The precision of the assay was ± 6%, and the accuracy was 96%. The final results were standardized and expressed as nanograms per milligram of creatinine (ng/mg Cr). Comparisons were made between study arms, based on infusions at cycles 4 and 6 using two sample t-test.

# REFERENCES

1. Tannock IF, de Wit R, Berry WR, Horti J, Pluzanska A, Chi KN, et al. Docetaxel plus prednisone or mitoxantrone plus prednisone for advanced prostate cancer. N Engl J Med. 2004;351:1502–12.

2. Cella D, Nichol MB, Eton D, Nelson JB, Mulani P. Estimating clinically meaningful changes for the Functional Assessment of Cancer Therapy--Prostate: results from a clinical trial of patients with metastatic hormone-refractory prostate cancer. Value Health. Elsevier BV; 2009;12:124–9.

3. Chen Q, Espey MG, Sun AY, Pooput C, Kirk KL, Krishna MC, et al. Pharmacologic doses of ascorbate act as a prooxidant and decrease growth of aggressive tumor xenografts in mice. Proceedings of the National Academy of Sciences. 2008;105:11105–9.

4. Ebenuwa I, Violet P-C, Padayatty S, Wang Y, Wang Y, Sun H, et al. Abnormal urinary loss of vitamin C in diabetes: prevalence and clinical characteristics of a vitamin C renal leak. Am J Clin Nutr. 2022;116:274–84.

5. Milne GL, Gao B, Terry ES, Zackert WE, Sanchez SC. Measurement of F2- isoprostanes and isofurans using gas chromatography-mass spectrometry. Free Radic Biol Med. 2013;59:36–44.
