# Supplementary material for: High-Dose Intravenous Vitamin C Combined with Docetaxel in Men with Metastatic Castration-Resistant Prostate Cancer: A Randomized Placebo-Controlled Phase II Trial
Source: Cancer Res Commun. 2024 Aug 20;4(8):2174–82. doi: 10.1158/2767-9764.CRC-24-0225 (PMC11333993; doi:10.1158/2767-9764.CRC-24-0225)
Supplement: Table S1 — shows Participating Sites and the Number of Patients Enrolled in the Trial at Each Institution [file crc-24-0225_table_s1_supps1.docx]

## Table S1. Participating Sites and the Number of Patients Enrolled in the Trial at Each Institution

| Site | Number of patients  docetaxel + HDIVC arm | | Number of patients  docetaxel + placebo arm |
| --- | --- | --- | --- |
| Johns Hopkins University | 5 | 1 | |
| Sibley Memorial Hospital | 8 | 3 | |
| Anne Arundel HSRI | 2 | 1 | |
| Karmanos Cancer Institute | 7 | 4 | |
| Seidman Cancer Center | 8 | 5 | |
| Thomas Jefferson University Hospital | 2 | 1 | |
