# Supplementary material for: High-Dose Intravenous Vitamin C Combined with Docetaxel in Men with Metastatic Castration-Resistant Prostate Cancer: A Randomized Placebo-Controlled Phase II Trial
Source: Cancer Res Commun. 2024 Aug 20;4(8):2174–82. doi: 10.1158/2767-9764.CRC-24-0225 (PMC11333993; doi:10.1158/2767-9764.CRC-24-0225)
Supplement: Table S2 — shows Association of HDIVC Treatment with PSA Response Adjusting for Prior Docetaxel Exposure [file crc-24-0225_table_s2_supps2.docx]

Table S2. Association of HDIVC Treatment with PSA Response Adjusting for Prior Docetaxel Exposure: The Breslow-Day test assessing the homogeneity of odds ratios yielded a non-significant result (p=0.32), indicating that the assumption of the CMH test statistic is met, and there are no differences in the odds ratios within the subsets categorized by prior therapy. It is worth noting that the power to detect an interaction of this nature is notably low, given the limited sample size of only ten patients who had received prior docetaxel. The stratum-specific odds ratios for PSA response in the HDIVC arm compared to the placebo arm can be found in footnotes b and c. The CMH test, assessing the combined odds ratio, did not reach statistical significance (p=0.72), with an estimated common odds ratio of 1.26 (95% CI: 0.29, 5.91). This result closely aligns with the unadjusted analysis, where the odds ratio is 1.37 (95% CI: 0.38, 4.94) (p= 0.63).

| Docetaxel | HDIVC | Number of participants with no PSA response | Number of participants with PSA response |
| --- | --- | --- | --- |
| No | No | 7 | 3 |
| No | Yes | 15 | 12 |
| Yes | No | 3 | 2 |
| Yes | Yes | 4 | 1 |

Common OR = 1.26 [CI: 0.29, 5.91] p value = 0.76

No Docetaxel OR = 1.87 [CI: 0.4, 8.8]

Yes Docetaxel OR = 0.38 [CI: 0.02, 6.35]
