# Supplementary material for: High-Dose Intravenous Vitamin C Combined with Docetaxel in Men with Metastatic Castration-Resistant Prostate Cancer: A Randomized Placebo-Controlled Phase II Trial
Source: Cancer Res Commun. 2024 Aug 20;4(8):2174–82. doi: 10.1158/2767-9764.CRC-24-0225 (PMC11333993; doi:10.1158/2767-9764.CRC-24-0225)
Supplement: Table S3 — shows Adverse Events Included in the Co-Primary Endpoint: All Occurrences [file crc-24-0225_table_s3_supps3.docx]

**Table S3. Adverse Events Included in the Co-Primary Endpoint: All Occurrences:** The frequencies of all adverse events that were specified by the co-primary objective. The table includes multiple AEs per patient.

|  | Treatment arm | Docetaxel + HDIVC | | Docetaxel + placebo | |
| --- | --- | --- | --- | --- | --- |
| Adverse event | Grade groups | 1-2 | 3-4 | 1-2 | 3-4 |
| Anorexia |  | 9 | 0 | 1 | 0 |
| Bone pain |  | 2 | 0 | 2 | 0 |
| Fatigue |  | 9 | 1 | 5 | 0 |
| Nausea |  | 2 | 1 | 1 | 0 |
| Total |  | 22 | 2 | 9 | 0 |
