# Supplementary material for: High-Dose Intravenous Vitamin C Combined with Docetaxel in Men with Metastatic Castration-Resistant Prostate Cancer: A Randomized Placebo-Controlled Phase II Trial
Source: Cancer Res Commun. 2024 Aug 20;4(8):2174–82. doi: 10.1158/2767-9764.CRC-24-0225 (PMC11333993; doi:10.1158/2767-9764.CRC-24-0225)
Supplement: Table S4 — shows Adverse Events Included in the Co-primary Endpoint: Worst Grade by Patient [file crc-24-0225_table_s4_supps4.docx]

**Table S4. Adverse Events Included in the Co-primary Endpoint: Worst Grade by Patient:** The proportion (and frequencies) of the worst grade adverse event of the specified types. The table includes one AE per patient. The one-sided Cochran Armitage trend test is 0.9.

| Treatment arm | Docetaxel + HDIVC  (n=32) | Docetaxel + placebo  (n=15) | Combined  (n=47) |
| --- | --- | --- | --- |
| Grade groups |  |  |  |
| 0 | 25% (8) | 40% (6) | 30% (14) |
| 1-2 | 69% (22) | 60% (9) | 66% (31) |
| 3-4 | 6% (2) | 0% (0) | 4% (2) |
