# Supplementary material for: High-Dose Intravenous Vitamin C Combined with Docetaxel in Men with Metastatic Castration-Resistant Prostate Cancer: A Randomized Placebo-Controlled Phase II Trial
Source: Cancer Res Commun. 2024 Aug 20;4(8):2174–82. doi: 10.1158/2767-9764.CRC-24-0225 (PMC11333993; doi:10.1158/2767-9764.CRC-24-0225)
Supplement: Table S5 — shows AEs with Attribution Possible, Probable or Definite to Treatment (HDVIC or Placebo) listed by types and grades [file crc-24-0225_table_s5_supps5.docx]

**Table S5. AEs with Attribution Possible, Probable or Definite to Treatment (HDVIC or Placebo) listed by types and grades**

| **Adverse event** | **Grade groups 1-2** | **Grade groups 3-4** |
| --- | --- | --- |
| Abdominal pain | 2 | 0 |
| Alkaline phosphatase increased | 0 | 1 |
| Alopecia | 5 | 0 |
| Anemia | 1 | 0 |
| Anorexia | 4 | 0 |
| Arthralgia | 1 | 0 |
| Bloating | 2 | 0 |
| Bone pain | 3 | 0 |
| Bruising | 2 | 0 |
| Chills | 2 | 0 |
| Chronic kidney disease | 1 | 1 |
| Constipation | 3 | 0 |
| Cough | 1 | 0 |
| Depression | 1 | 0 |
| Diarrhea | 7 | 2 |
| Dizziness | 6 | 0 |
| Dry mouth | 5 | 0 |
| Dysgeusia | 5 | 0 |
| Dysphagia | 1 | 0 |
| Edema face | 1 | 0 |
| Edema limbs | 3 | 0 |
| Fall | 1 | 0 |
| Fatigue | 13 | 0 |
| Fever | 1 | 0 |
| Flushing | 3 | 0 |
| Generalized muscle weakness | 2 | 0 |
| Headache | 7 | 0 |
| Hot flashes | 1 | 0 |
| Hypertension | 0 | 2 |
| Hypoalbuminemia | 1 | 0 |
| Hypocalcemia | 1 | 0 |
| Hypoglycemia | 0 | 1 |
| Hypokalemia | 1 | 1 |
| Hyponatremia | 2 | 0 |
| Hypophosphatemia | 0 | 1 |
| Hypotension | 1 | 0 |
| Infections and infestations | 1 | 0 |
| Infusion site extravasation | 1 | 0 |
| Lymphocyte count decreased | 2 | 0 |
| Malaise | 6 | 0 |
| Mucositis oral | 1 | 0 |
| Nail discoloration | 2 | 0 |
| Nausea | 8 | 1 |
| Neck pain | 1 | 0 |
| Nervous system disorders | 1 | 0 |
| Neutrophil count decreased | 0 | 1 |
| Oral pain | 1 | 0 |
| Pain | 2 | 0 |
| Pain in extremity | 3 | 0 |
| Paresthesia | 1 | 0 |
| Peripheral sensory neuropathy | 2 | 0 |
| Platelet count decreased | 1 | 0 |
| Pruritus | 1 | 0 |
| Rash maculo-papular | 2 | 0 |
| Sinus tachycardia | 2 | 0 |
| Upper respiratory infection | 1 | 0 |
| Urinary incontinence | 1 | 0 |
| Urinary tract infection | 1 | 0 |
| Vomiting | 4 | 0 |
| Weight loss | 2 | 0 |
| Sum | 137 | 11 |
