# Supplementary material for: High-Dose Intravenous Vitamin C Combined with Docetaxel in Men with Metastatic Castration-Resistant Prostate Cancer: A Randomized Placebo-Controlled Phase II Trial
Source: Cancer Res Commun. 2024 Aug 20;4(8):2174–82. doi: 10.1158/2767-9764.CRC-24-0225 (PMC11333993; doi:10.1158/2767-9764.CRC-24-0225)
Supplement: Table S6 — shows AEs with Attribution Possible, Probable or Definite to Docetaxel listed by types and grades [file crc-24-0225_table_s6_supps6.docx]

**Table S6. AEs with Attribution Possible, Probable or Definite to Docetaxel** **listed by types and grades**

| **Adverse event** | | **Grade groups 1-2** | **Grade groups 3-4** | |
| --- | --- | --- | --- | --- |
| Abdominal pain | 3 | | | 0 |
| Acute kidney injury | 0 | | | 1 |
| Alanine aminotransferase increased | 1 | | | 0 |
| Alkaline phosphatase increased | 2 | | | 1 |
| Allergic reaction | 1 | | | 0 |
| Allergic rhinitis | 2 | | | 0 |
| Alopecia | 20 | | | 0 |
| Anal mucositis | 1 | | | 0 |
| Anemia | 11 | | | 3 |
| Anorexia | 13 | | | 0 |
| Arthralgia | 2 | | | 0 |
| Bloating | 1 | | | 0 |
| Blurred vision | 1 | | | 0 |
| Bone pain | 6 | | | 0 |
| Bronchial infection | 1 | | | 0 |
| Bruising | 3 | | | 0 |
| Cardiac disorders | 1 | | | 0 |
| Chills | 6 | | | 0 |
| Chronic kidney disease | 2 | | | 1 |
| Concentration impairment | 1 | | | 0 |
| Constipation | 7 | | | 0 |
| Cough | 2 | | | 0 |
| Creatinine increased | 1 | | | 1 |
| Dehydration | 1 | | | 1 |
| Depression | 1 | | | 0 |
| Diarrhea | 23 | | | 2 |
| Dizziness | 7 | | | 0 |
| Dry mouth | 5 | | | 0 |
| Dry skin | 1 | | | 0 |
| Dysgeusia | 8 | | | 0 |
| Dysphagia | 2 | | | 0 |
| Dyspnea | 4 | | | 0 |
| Edema face | 1 | | | 0 |
| Edema limbs | 8 | | | 0 |
| Epistaxis | 2 | | | 0 |
| Eye disorders | 2 | | | 0 |
| Fall | 1 | | | 0 |
| Fatigue | 24 | | | 1 |
| Febrile neutropenia | 0 | | | 7 |
| Fever | 3 | | | 1 |
| Flu like symptoms | 1 | | | 0 |
| Flushing | 4 | | | 0 |
| Gait disturbance | 1 | | | 0 |
| General disorders and administration site condition | 1 | | | 0 |
| Generalized muscle weakness | 5 | | | 0 |
| Headache | 7 | | | 0 |
| Hot flashes | 1 | | | 0 |
| Hyperglycemia | 3 | | | 0 |
| Hypernatremia | 1 | | | 0 |
