# Supplementary material for: High-Dose Intravenous Vitamin C Combined with Docetaxel in Men with Metastatic Castration-Resistant Prostate Cancer: A Randomized Placebo-Controlled Phase II Trial
Source: Cancer Res Commun. 2024 Aug 20;4(8):2174–82. doi: 10.1158/2767-9764.CRC-24-0225 (PMC11333993; doi:10.1158/2767-9764.CRC-24-0225)
Supplement: Table S7 — shows Serious Adverse Events (N = 11 patients), by Type and Grade. Attributions Assigned Docetaxel versus HDIVC or Placebo [file crc-24-0225_table_s7_supps7.docx]

**Table S7. Serious Adverse Events (N=11 patients), by Type and Grade. Attributions Assigned Docetaxel versus HDIVC or Placebo**

| **Serious Adverse Event** | **Grade** | **HDIVC/Placebo** | **Docetaxel** |
| --- | --- | --- | --- |
| Neutrophil count decreased | 4 | Unrelated | Definite |
| Abdominal pain | 1 | Unrelated | Unrelated |
| Alanine aminotransferase increased | 1 | Unrelated | Unrelated |
| Aspartate aminotransferase increased | 1 | Unrelated | Unrelated |
| Back pain | 1 | Unrelated | Unrelated |
| Fall | 3 | Unrelated | Unrelated |
| Febrile neutropenia | 3 | Unrelated | Definite |
| Hematuria | 3 | Unrelated | Unrelated |
| Leukocytosis | 2 | Unrelated | Definite |
| Urinary tract infection | 3 | Unrelated | Definite |
| Febrile neutropenia | 3 | Unrelated | Definite |
| Dyspnea | 3 | Unrelated | Unlikely |
| Fever | 3 | Unrelated | Possible |
| Infections and infestations - Other specify | 3 | Unrelated | Possible |
| Investigations - Other specify | 3 | Unrelated | Unrelated |
| Abdominal pain | 3 | Unrelated | Unrelated |
| Cholecystitis | 3 | Unrelated | Unrelated |
| Lung infection | 3 | Unrelated | Unrelated |
| Febrile neutropenia | 3 | Unrelated | Definite |
| Atrial fibrillation | 3 | Unrelated | Unrelated |
| Febrile neutropenia | 4 | Unrelated | Unrelated |
| Anorexia | 2 | Unrelated | Unrelated |
| Dehydration | 4 | Unrelated | Unrelated |
| Investigations - Other specify | 2 | Unrelated | Unrelated |
| Investigations - Other specify | 3 | Unrelated | Unrelated |
| Investigations - Other specify | 4 | Unrelated | Unrelated |
| Myocardial infarction | 2 | Unrelated | Unrelated |
