# Supplementary material for: High-Dose Intravenous Vitamin C Combined with Docetaxel in Men with Metastatic Castration-Resistant Prostate Cancer: A Randomized Placebo-Controlled Phase II Trial
Source: Cancer Res Commun. 2024 Aug 20;4(8):2174–82. doi: 10.1158/2767-9764.CRC-24-0225 (PMC11333993; doi:10.1158/2767-9764.CRC-24-0225)
Supplement: Table S8 — shows Prevalence of adverse events [file crc-24-0225_table_s8_supps8.docx]

Table S8. Prevalence of adverse events for safety analysis set plus one patient randomized but not treated. For patients with repeated events of the same toxicity, the earliest instance of the AE with the highest grade and attribution combination is reported. D+A (red) Docetaxel + HDIVC, and D+P (blue) Docetaxel + Placebo.

**
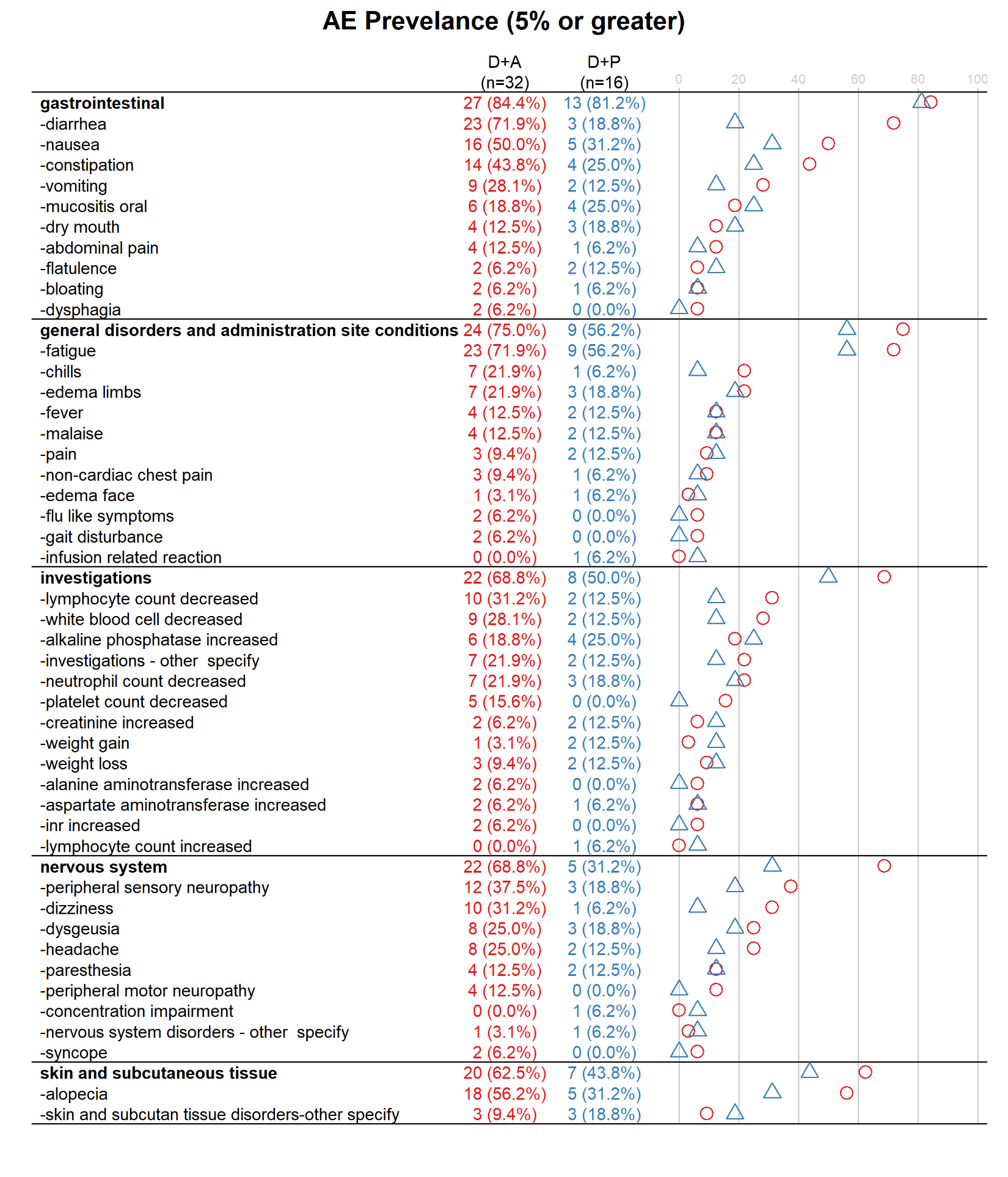
**

**
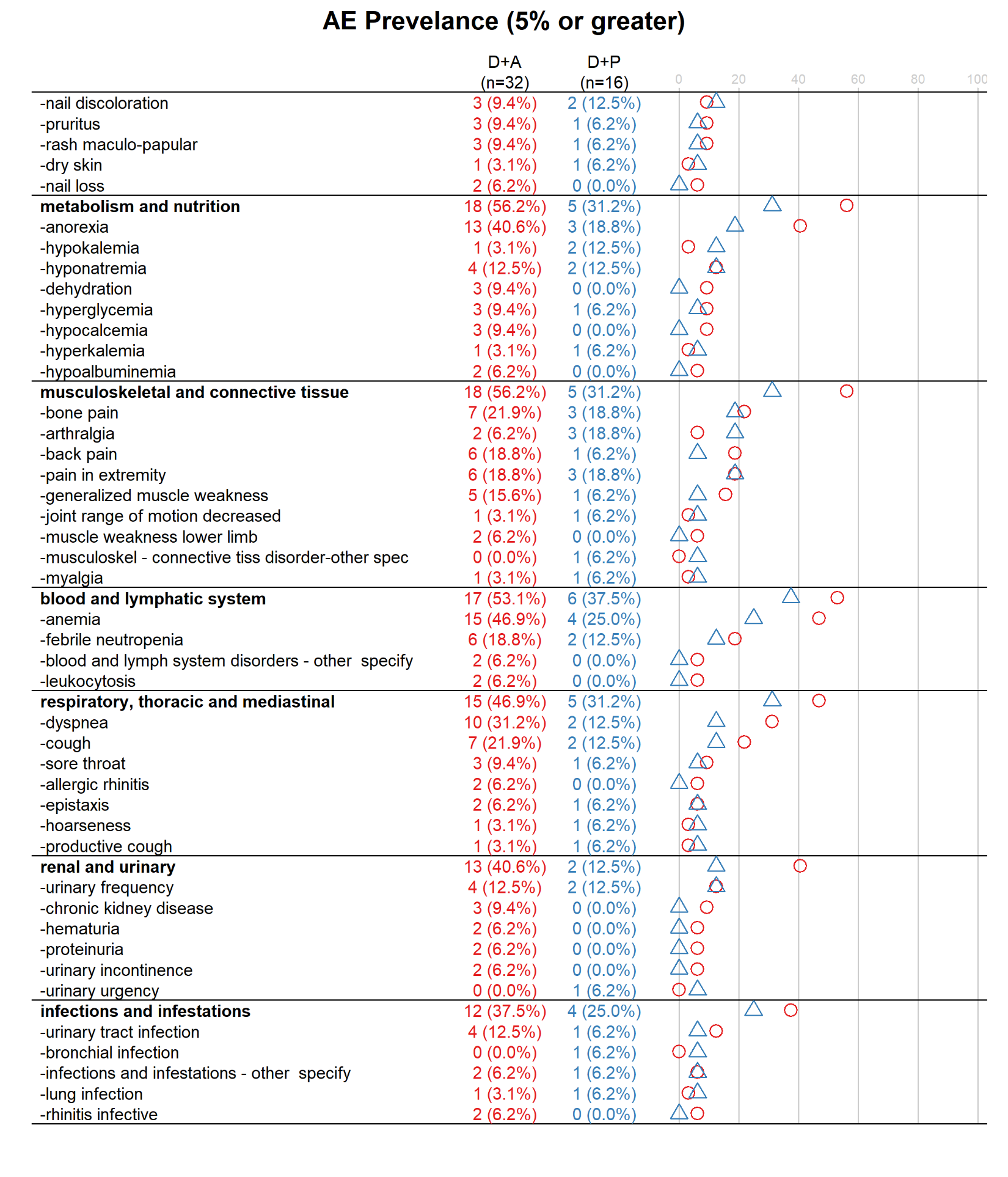
**

**
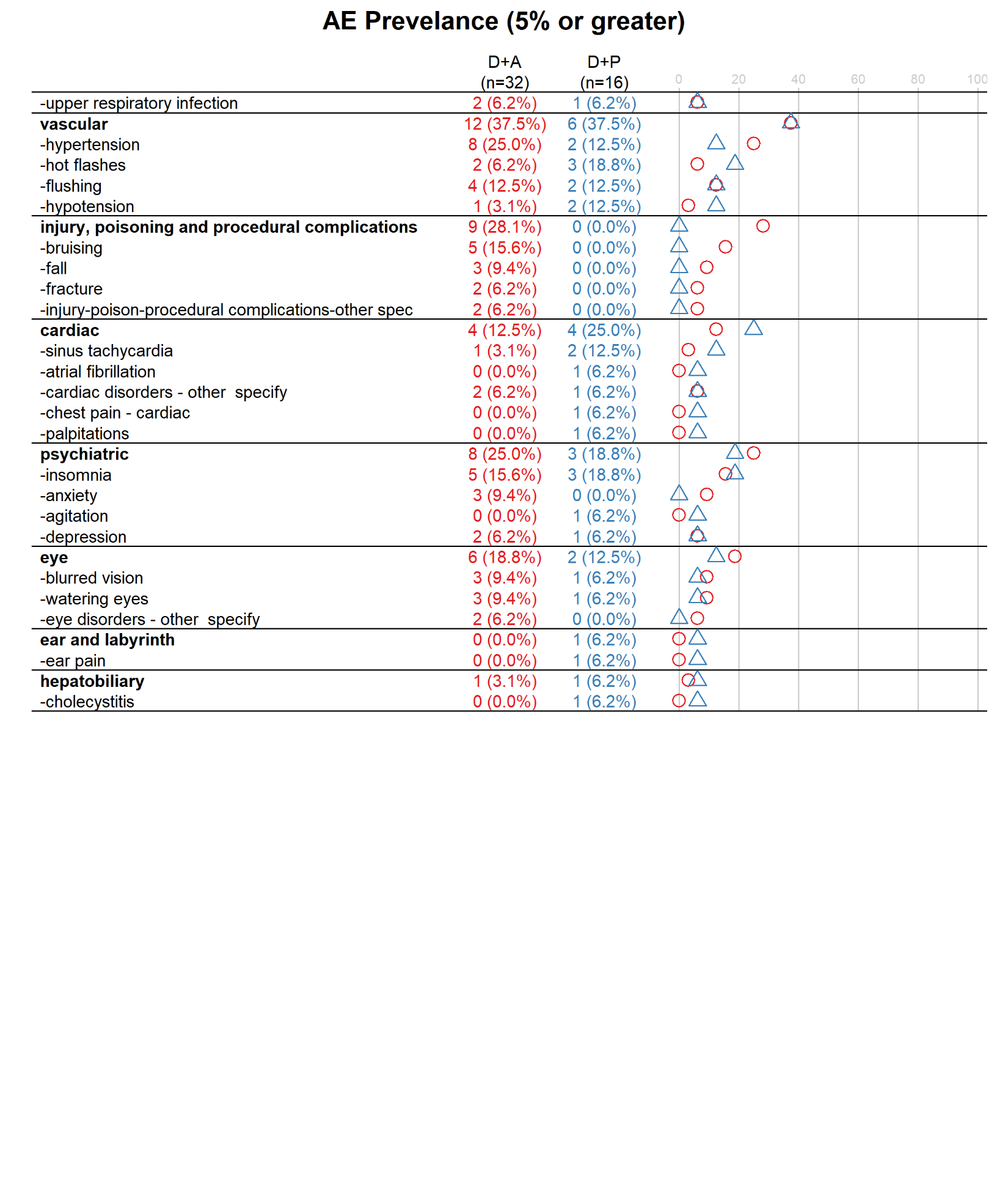
**
