# Supplementary material for: High-Dose Intravenous Vitamin C Combined with Docetaxel in Men with Metastatic Castration-Resistant Prostate Cancer: A Randomized Placebo-Controlled Phase II Trial
Source: Cancer Res Commun. 2024 Aug 20;4(8):2174–82. doi: 10.1158/2767-9764.CRC-24-0225 (PMC11333993; doi:10.1158/2767-9764.CRC-24-0225)
Supplement: Table S9 — shows FACT-P Total Scores: Baseline, Cycles 4, 6, and 8 [file crc-24-0225_table_s9_supps9.docx]

**Table S9. FACT-P Total Scores: Baseline (BL), Cycles 4, 6, and 8 (C4, C6, C8):** Summary statistics by treatment arm, medians and ranges. Higher FACT-P scores indicate a better QoL. Baseline scores were obtained for 42 of 47 patients (89%). Patient pairwise scores at all time points were highly correlated, with Pearson correlation coefficients 0.75 or greater

| Variable | n | missing | Docetaxel + HDIVC | Docetaxel + placebo |
| --- | --- | --- | --- | --- |
| BLscore, Median (min, max) | 42 | 5 | 113 (82, 150) | 112 (52.2, 149) |
| C4score, Median (min, max) | 29 | 18 | 114 (84, 150) | 130 (77.4, 142) |
| C6score, Median (min, max) | 24 | 23 | 105 (68, 145) | 110 (76, 127) |
| C8score, Median (min, max) | 16 | 31 | 108 (79, 145) | 101 (74.7, 123) |

Note: Table includes all patients randomized (N=50)
