# Supplementary material for: High-Dose Intravenous Vitamin C Combined with Docetaxel in Men with Metastatic Castration-Resistant Prostate Cancer: A Randomized Placebo-Controlled Phase II Trial
Source: Cancer Res Commun. 2024 Aug 20;4(8):2174–82. doi: 10.1158/2767-9764.CRC-24-0225 (PMC11333993; doi:10.1158/2767-9764.CRC-24-0225)
Supplement: Table S10 — shows ANCOVA Least-squares Means for Cycle 4, 6, and 8 FACT-P Total by Study Arm [file crc-24-0225_table_s10_supps10.docx]

**Table S10. ANCOVA Least-squares Means for Cycle 4, 6, and 8 FACT-P Total by Study Arm:** The analysis plan calls for ANCOVA of the FACT-P total scores for the three on-study time points, adjusting for baseline FACT-P score.

| Treatment | Ismean | SE | df | Lower CL | Upper CL |
| --- | --- | --- | --- | --- | --- |
| Cycle 4 |  |  |  |  |  |
| Docetaxel+HDIVC | 116.2996 | 2.7106 | 24 | 110.7052 | 121.8940 |
| Docetaxel+Placebo | 114.0941 | 4.1837 | 24 | 105.4593 | 122.7289 |
| Cycle 6 |  |  |  |  |  |
| Docetaxel+HDIVC | 108.1332 | 3.6028 | 20 | 100.6179 | 115.6485 |
| Docetaxel+Placebo | 108.1099 | 5.4498 | 20 | 96.7417 | 119.4781 |
| Cycle 8 |  |  |  |  |  |
| Docetaxel+HDIVC | 107.8677 | 3.3378 | 13 | 100.6567 | 115.0787 |
| Docetaxel+Placebo | 106.7207 | 5.1162 | 13 | 95.6679 | 117.7735 |

Confidence level used: 0.95. Confidence interval widths have not been adjusted for multiplicity and may not be used in place of hypothesis testing.
