# Supplementary material for: High-Dose Intravenous Vitamin C Combined with Docetaxel in Men with Metastatic Castration-Resistant Prostate Cancer: A Randomized Placebo-Controlled Phase II Trial
Source: Cancer Res Commun. 2024 Aug 20;4(8):2174–82. doi: 10.1158/2767-9764.CRC-24-0225 (PMC11333993; doi:10.1158/2767-9764.CRC-24-0225)
Supplement: Table S11 — shows FACT-P Change (on Study Minus Baseline) Scores [file crc-24-0225_table_s11_supps11.docx]

**Table S11. FACT-P Change (on Study Minus Baseline) Scores:** In the analysis of FACT-P change scores, positive changes (on-study scores minus baseline scores) indicate improvements from the baseline measurements. Positive mean differences (docetaxel + HDIVC scores minus docetaxel + placebo scores) signify a treatment arm benefit.

|  | N | Docetaxel+HDIVC  N = 32 | Docetaxel+Placebo  N = 15 |
| --- | --- | --- | --- |
| C4chg | 27 | -1.8 2.0 8.2 (4.8 ±12.0) | -7.0 -2.1 6.0 (1.1 ±18.8) |
| C6chg | 23 | -10.18 1.25 7.30 (-0.34 ± 12.99) | -12.70 1.00 7.50 (0.50 ± 22.12) |
| C8chg | 16 | -6.8 -3.0 2.5 (-1.8 ±11.6) | -7.8 -2.7 8.0 (2.4 ±13.0) |

a b c represents the lower quartile a, the median b, and the upper quartile c for continuous variables.

x ± s represents X¯ ± 1 SD.

N is the number of non–missing values.
