# Supplementary material for: High-Dose Intravenous Vitamin C Combined with Docetaxel in Men with Metastatic Castration-Resistant Prostate Cancer: A Randomized Placebo-Controlled Phase II Trial
Source: Cancer Res Commun. 2024 Aug 20;4(8):2174–82. doi: 10.1158/2767-9764.CRC-24-0225 (PMC11333993; doi:10.1158/2767-9764.CRC-24-0225)
Supplement: Table S12 — shows Comparison of FACT-P Change Scores Between Study Arms: Mean Difference Docetaxel + HDVIC Minus Docetaxel + Placebo [file crc-24-0225_table_s12_supps12.docx]

**Table S12. Comparison of FACT-P Change Scores Between Study Arms: Mean Difference D+HDVIC Minus D+Placebo:** In the analysis of FACT-P change scores, positive changes (on-study scores minus baseline scores) indicate improvements from the baseline measurements. Positive mean differences (docetaxel + HDIVC scores minus docetaxel + placebo scores) signify a treatment arm benefit.

| Variable | *n*_D+HDIVC_ | x¯_D+HDIVC_ | *n*_D+P_ | x¯_D+P_ | mean difference | CI *t* |
| --- | --- | --- | --- | --- | --- | --- |
| C4.change | 19 | 4.85 | 8 | 1.12 | 3.73 | [-12.43, 19.89] |
| C6.change | 16 | -0.34 | 7 | 0.50 | -0.84 | [-21.57, 19.90] |
| C8.change | 11 | -1.77 | 5 | 2.40 | -4.17 | [-20.14, 11.81] |

Confidence level used: 0.95. Confidence interval widths have not been adjusted for multiplicity and may not be used in place of hypothesis testing
