# Supplementary material for: High-Dose Intravenous Vitamin C Combined with Docetaxel in Men with Metastatic Castration-Resistant Prostate Cancer: A Randomized Placebo-Controlled Phase II Trial
Source: Cancer Res Commun. 2024 Aug 20;4(8):2174–82. doi: 10.1158/2767-9764.CRC-24-0225 (PMC11333993; doi:10.1158/2767-9764.CRC-24-0225)
Supplement: Table S13 — shows Comparison of F2-Isoprostanes at Baseline between Treatment and Control Arms [file crc-24-0225_table_s13_supps13.docx]

**Table S13. Comparison of F2-Isoprostanes at Baseline between Treatment and Control Arms**

**Variable *n*_Control_** ***x*¯_Control_ *n*_HDIVC_ *x*¯_HDIVC_ mean difference CI *t***

| Iso8PGF 13 | 0.24 25 | 0.21 | 0.03 | [-0.03, 0.10] |
| --- | --- | --- | --- | --- |
| PGF2a 10 | 0.27 20 | 0.24 | 0.03 | [-0.09, 0.15] |
| Iso5F2t 10 | 0.42 20 | 0.29 | 0.14 | [-0.07, 0.34] |
| Iso5F2c 10 | 1.12 20 | 0.85 | 0.27 | [-0.10, 0.65] |

Confidence level used: 0.95. Confidence interval widths have not been adjusted for multiplicity and may not be used in place of hypothesis testing
