# Supplementary material for: High-Dose Intravenous Vitamin C Combined with Docetaxel in Men with Metastatic Castration-Resistant Prostate Cancer: A Randomized Placebo-Controlled Phase II Trial
Source: Cancer Res Commun. 2024 Aug 20;4(8):2174–82. doi: 10.1158/2767-9764.CRC-24-0225 (PMC11333993; doi:10.1158/2767-9764.CRC-24-0225)
Supplement: Table S14 — shows Comparison of F2-Isoprostanes Control and Intervention Changes (post - pre) Immediately after Cycle 4 [file crc-24-0225_table_s14_supps14.docx]

**Table S14. Control and Intervention Changes (post - pre) Immediately after Cycle 4**

**Variable  *n*_Control_ *x*¯Control *n*_HDIVC_ *x*¯_HDIVC_  mean difference**   **CI *t***

| Iso8PGF | 3 | 0.02 | 5 | 0.12 | -0.10 |  | [-0.36, 0.16] |
| --- | --- | --- | --- | --- | --- | --- | --- |
| PGF2a | 2 | -0.01 | 5 | 0.52 | -0.52 |  | [-1.27, 0.22] |
| Iso5F2t | 2 | 0.12 | 5 | 0.27 | -0.15 |  | [-1.02, 0.72] |
| Iso5F2c | 2 | 0.18 | 5 | 0.31 | -0.13 |  | [-1.37, 1.11] |

Confidence level used: 0.95. Confidence interval widths have not been adjusted for multiplicity and may not be used in place of hypothesis testing
