# Supplementary material for: High-Dose Intravenous Vitamin C Combined with Docetaxel in Men with Metastatic Castration-Resistant Prostate Cancer: A Randomized Placebo-Controlled Phase II Trial
Source: Cancer Res Commun. 2024 Aug 20;4(8):2174–82. doi: 10.1158/2767-9764.CRC-24-0225 (PMC11333993; doi:10.1158/2767-9764.CRC-24-0225)
Supplement: Table S15 — shows Comparison of F2-Isoprostanes Control and Intervention Changes (post - pre) Immediately after Cycle 6 [file crc-24-0225_table_s15_supps15.docx]

**Table S15. Control and Intervention Changes (post - pre) Immediately after Cycle 6**

**Variable *n*_Control_  *x*¯Control *n*_HDIVC_  *x*¯_HDIVC_ mean difference CI *t***

| Iso8PGF | 3 | -0.04 | 5 | 0.00 | -0.04 |  | [-0.18, 0.10] |
| --- | --- | --- | --- | --- | --- | --- | --- |
| PGF2a | 2 | -0.06 | 5 | 0.37 | -0.43 |  | [-1.17, 0.30] |
| Iso5F2t | 2 | -0.01 | 5 | -0.04 | 0.03 |  | [-0.26, 0.32] |
| Iso5F2c | 2 | -0.12 | 5 | -0.16 | 0.04 |  | [-0.69, 0.77] |

Confidence level used: 0.95. Confidence interval widths have not been adjusted for multiplicity and may not be used in place of hypothesis testing
