# Supplementary material for: High-Dose Intravenous Vitamin C Combined with Docetaxel in Men with Metastatic Castration-Resistant Prostate Cancer: A Randomized Placebo-Controlled Phase II Trial
Source: Cancer Res Commun. 2024 Aug 20;4(8):2174–82. doi: 10.1158/2767-9764.CRC-24-0225 (PMC11333993; doi:10.1158/2767-9764.CRC-24-0225)
Supplement: Table S16 — shows Comparison of F2-Isoprostanes Control and Intervention Changes 60 minutes after Cycle 4 [file crc-24-0225_table_s16_supps16.docx]

**Table S16. Control and Intervention Changes 60 Minutes after Cycle 4**

**Variable *n*_Control_  *x*¯Control *n*_HDIVC_ *x*¯_HDIVC_ mean difference CI *t***

| Iso8PGF | 4 | -0.02 | 5 | 0.11 | -0.13 |  | [-0.27, 0.02] |
| --- | --- | --- | --- | --- | --- | --- | --- |
| PGF2a | 3 | -0.03 | 5 | 0.24 | -0.28 |  | [-0.58, 0.03] |
| Iso5F2t | 3 | 0.04 | 5 | 0.23 | -0.19 |  | [-0.45, 0.07] |
| Iso5F2c | 3 | 0.01 | 5 | 0.31 | -0.30 |  | [-0.78, 0.19] |

Confidence level used: 0.95. Confidence interval widths have not been adjusted for multiplicity and may not be used in place of hypothesis testing
