# Supplementary material for: High-Dose Intravenous Vitamin C Combined with Docetaxel in Men with Metastatic Castration-Resistant Prostate Cancer: A Randomized Placebo-Controlled Phase II Trial
Source: Cancer Res Commun. 2024 Aug 20;4(8):2174–82. doi: 10.1158/2767-9764.CRC-24-0225 (PMC11333993; doi:10.1158/2767-9764.CRC-24-0225)
Supplement: Table S17 — shows Comparison of F2-Isoprostanes Control and Intervention Changes 60 minutes after Cycle 6 [file crc-24-0225_table_s17_supps17.docx]

**Table S17. Control and Intervention Changes 60 Minutes after Cycle 6**

**Variable *n*_Control_  *x*¯Control *n*_HDIVC_  *x*¯_HDIVC_ mean difference CI *t***

| Iso8PGF | 4 | 0.00 | 4 | -0.00 | 0.00 |  | [-0.20, 0.20] |
| --- | --- | --- | --- | --- | --- | --- | --- |
| PGF2a | 3 | 0.01 | 4 | 0.28 | -0.27 |  | [-0.89, 0.35] |
| Iso5F2t | 3 | 0.09 | 4 | -0.05 | 0.14 |  | [-0.26, 0.53] |
| Iso5F2c | 3 | 0.18 | 4 | -0.19 | 0.38 |  | [-0.44, 1.19] |

Confidence level used: 0.95. Confidence interval widths have not been adjusted for multiplicity and may not be used in place of hypothesis testing
