# Supplementary material for: High-Dose Intravenous Vitamin C Combined with Docetaxel in Men with Metastatic Castration-Resistant Prostate Cancer: A Randomized Placebo-Controlled Phase II Trial
Source: Cancer Res Commun. 2024 Aug 20;4(8):2174–82. doi: 10.1158/2767-9764.CRC-24-0225 (PMC11333993; doi:10.1158/2767-9764.CRC-24-0225)

**Figure S1. FACT-P score: A.** **FACT-P Score Changes in the Control Group by Cycle and Subject. B. FACT-P Score Changes in the Treatment Group by Cycle and Subject. C. Total FACT-P Score in the Control Group by Cycle and Subject. D. Total FACT-P Score in the Treatment Group by Cycle and Subject. E. Correlations and ANCOVA Plots by Cycle.**

**A.**


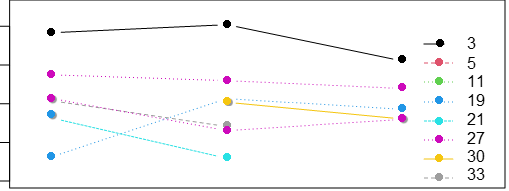


**FACT-P Change in Score**

**-40 -20 0 20 40**

1. 6 8

**B.**


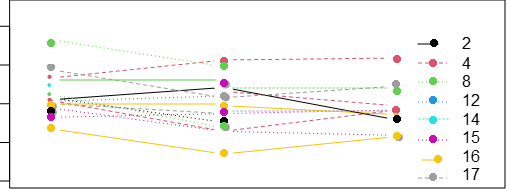


**FACT-P Change in Score**

**-40 -20 0 20 40**

1. 6 8

**C.**


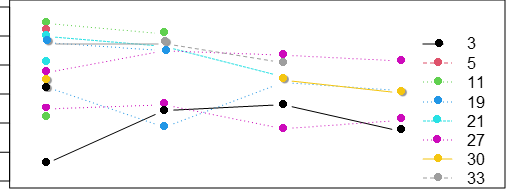


**FACT-P Score**

**40 80 120 160**

1 4 6 8

**D.**


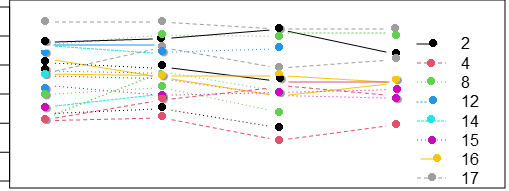


**FACT-P Score**

**40 80 120 160**

1 4 6 8

**E.**


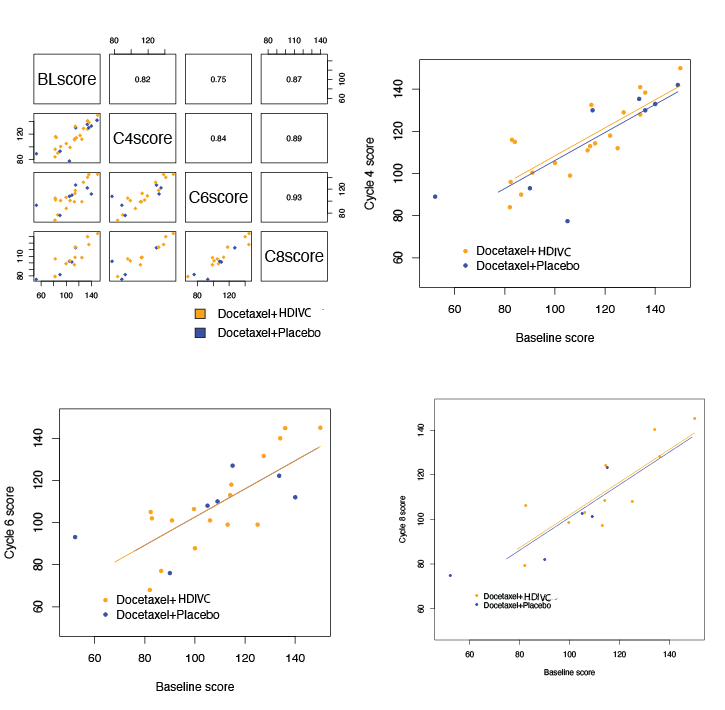

Supplement: Figure S1 — shows FACT-P scores [file crc-24-0225_figure_s1_suppsf1.docx]
