# Supplementary material for: High-Dose Intravenous Vitamin C Combined with Docetaxel in Men with Metastatic Castration-Resistant Prostate Cancer: A Randomized Placebo-Controlled Phase II Trial
Source: Cancer Res Commun. 2024 Aug 20;4(8):2174–82. doi: 10.1158/2767-9764.CRC-24-0225 (PMC11333993; doi:10.1158/2767-9764.CRC-24-0225)

Figure S2. Plasma ascorbic acid concentrations (uM) in seven subjects before infusion, at 60 minutes of infusion, and at the end of infusion. Error bars (standard deviations) indicate measurements from samples obtained on different infusion days from the same participants. Pre-infusion values are consistent with those expected in humans.


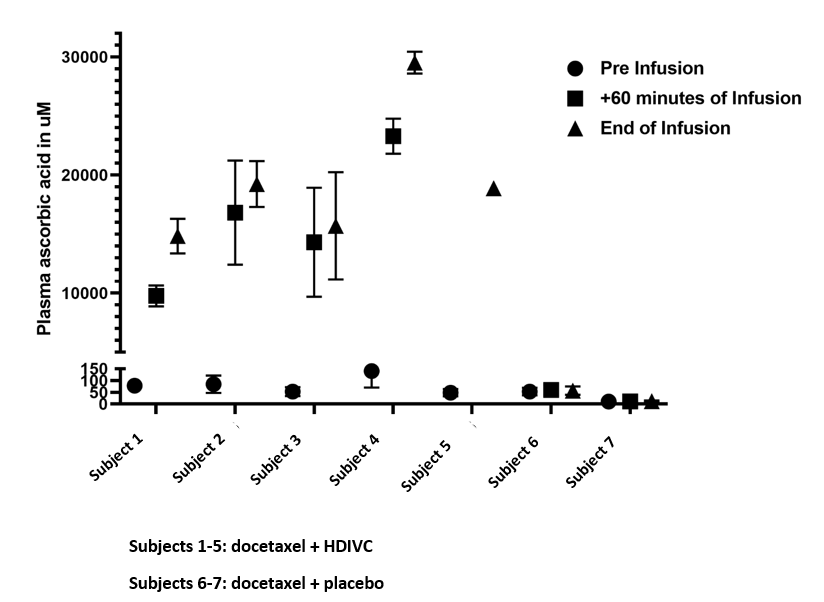

Supplement: Figure S2 — shows Plasma ascorbic acid concentrations [file crc-24-0225_figure_s2_suppsf2.docx]
