# Supplementary material for: High-Dose Intravenous Vitamin C Combined with Docetaxel in Men with Metastatic Castration-Resistant Prostate Cancer: A Randomized Placebo-Controlled Phase II Trial
Source: Cancer Res Commun. 2024 Aug 20;4(8):2174–82. doi: 10.1158/2767-9764.CRC-24-0225 (PMC11333993; doi:10.1158/2767-9764.CRC-24-0225)
Supplement: Supplementary: Trial Protocol — Trial Protocol shows the details of the study [file crc-24-0225_supplementary_trial_protocol_suppst.pdf]

A Randomized Phase 2 Trial of Ascorbic Acid in Combination with Docetaxel in Men with Metastatic Prostate Cancer

Protocol ID: J15106, IRB00070691

Principal Investigator:
Channing Paller, MD
Johns Hopkins University School of Medicine
Sidney Kimmel Comprehensive Cancer Center
201 N. Broadway, Viragh 9162
Baltimore, MD 21287
Phone: (410) 955-8239 (Office) or 202-660-6322 (Clinic)
Fax: 410-614-8397
Email: cpaller1@jhmi.edu

Statistician:
Marianna Zahurak, MS
410-955-4219
zahurma@jhmi.edu

Lead Study Coordinator:
Amber Michalik
amichal2@jhmi.edu

Responsible Research Nurse:
Kathleen Schultz, MS
410-614-9482
kschult3@jhmi.edu

Responsible Regulatory Manager:
Jennifer Durham, PhD
410-502-3038/ (F) 410-614-8216
juram1@jhmi.edu

Coordinating Center:
Johns Hopkins University School of Medicine
Sidney Kimmel Comprehensive Cancer Center
201 N. Broadway, Viragh Floor 9
Baltimore, MD 21287
crocc@jhmi.edu

IND Sponsor: **Channing Paller, MD**

IND: IND 126543

Investigational Agent: Ascorbic Acid (McGuff Pharmaceuticals Inc.)

Commercial Agent: Docetaxel

Participating Sites:

- Barbara Ann Karmanos Cancer Institute
  Site PI: Elisabeth I. Heath, MD FACP
  Professor of Oncology and Medicine
  Director, Prostate Cancer Research
  Karmanos Cancer Institute
  Wayne State University School of Medicine
  4100 John R, HW04H0
  Detroit, MI 48201
  Office: 313-576-8715
  Fax: 313-576-8767
  Email: heathe@karmanos.org

- Thomas Jefferson University
  Site PI: William K. Kelly, DO
  Thomas Jefferson University
  925 Chestnut St. Suite 320A
  Philadelphia, PA 19107
  Office: 215-503-4490
  Fax: 215-503-3408
  Email: William.Kelly@jefferson.edu

- University Hospitals of Cleveland
  Seidman Cancer Center
  PI: Prateek Mendiratta, MD
  University Hospitals of Cleveland
  11100 Euclid Ave
  Cleveland, OH 44106
  Office: 216-844-6031

  Fax: 216-844-1721
  Email: Prateek.mendiratta@uhhospitals.org

- Anne Arundel Health System, Research Institute
  PI: Imad Tabbara, MD

2003 Medical Parkway, Wayson Pavilion Ste. 210
Annapolis, MD 21401
Office: 443-481-4884
Fax: 443-481-4893
Email: itabbara@aahs.org

Protocol Type / Version # / Version Date:

Original/Version 1.0/ April 30, 2015
Original/Version 1.1/ May 25, 2015
Original/Version 1.2/ June 03, 2015
Original/Version 1.3/ July 06, 2015
Original/Version 1.4/September 28, 2015
Protocol Version 2.0/ Date December 21, 2015
Protocol Version 3.0/Date February 4, 2016
Protocol Version 4.0/Date May 4, 2016
Protocol Version 5.0/Date August 12, 2016
Protocol Version 6.0/Date September 1, 2016
Protocol Version 7.0/Date March 3, 2017
Protocol Version 8.0/Date May 10, 2017
Protocol Version 9.0/Date July 27, 2017
Protocol Version 10.0/Date: August 31, 2017
Protocol Version 11.0/Date: November 3, 2017
Protocol Version 12.0/Date: January 30, 2018
Protocol Version 13.0/Date: June 11, 2019
Protocol Version 14.0/Date: October 9, 2019
Protocol Version 15.0/Date: July 1, 2020
Protocol Version 16.0/Date: Sept. 17, 2020
Protocol Version 17.0/Date: Nov. 17, 2020
Protocol Version 18.0/Date: Oct. 19, 2021
Protocol Version 19.0/Date: Aug 16, 2022

SCHEMA

Bone and CT scans at Baseline, every 12 weeks and at the end of study

Visits and labs on Day 1 of 8 Cycles (Cycle length is 21 days)

Randomize

Arm A, n=42
Docetaxel (75 mg/m^2, Q3 weeks) and IV Ascorbic acid (1g/kg, 2 times per week)
(First three doses (over 1.5 weeks): titrate 0.25 g/kg, 0.5 g/kg, and 0.75 mg/kg. Starting with Cycle 1 Week 2, the dose of Ascorbate will be 1 g/kg as long as this dose is tolerated.)

Arm B, n=21
Docetaxel (75 mg/m^2, Q3 weeks) and Placebo (2 times per week)

TABLE OF CONTENTS

Page

SCHEMA iv

1. OBJECTIVES 1
1.1 Primary Objectives 1
1.2 Secondary Objectives 1
1.3 Exploratory Objectives 1
1.4 Study Design 1

2. BACKGROUND 2
2.1 Prostate cancer 2
2.2 Docetaxel (Taxotere) in Prostate Cancer 2
2.3 Clinical Efficacy and Safety of I.V. Ascorbic Acid In Cancer Patients 3
2.4 Ascorbic Acid Mechanism of Action in Prostate Cancer 4
2.5 Rationale for Combination Therapy With Ascorbic Acid and Chemotherapy 5
2.6 Justification for Using IV Ascorbic Acid with Docetaxel Prostate Cancer at Johns Hopkins 5

3. PATIENT SELECTION 6
3.1 Eligibility Criteria 6
3.2 Exclusion Criteria 7

4. REGISTRATION PROCEDURES 8
4.1 General Guidelines 8
4.2 Registration Process 8
4.3 Multicenter Guidelines 9

5. TREATMENT PLAN 10
5.1 Agent Administration 10
5.3 General Concomitant Medication and Supportive Care Guidelines 11
5.4 Expected Adverse Events 11
5.5 Duration of Therapy and Criteria for Removal from Study 12
5.6 Duration of Follow Up 14

6. DOSING DELAYS/DOSE MODIFICATIONS 14
6.1 Dosing Delays 14
6.2 Dose Modifications 16

7. ADVERSE EVENTS: LIST AND REPORTING REQUIREMENTS 16
7.1 Definitions 17
7.2 Relationship 18
7.3 Expectedness 18
7.4 Handling of Expedited Safety Reports 19
7.5 Reporting 19

8. PHARMACEUTICAL INFORMATION 21
8.1 Ascorbic acid 21

8.2 Docetaxel (Taxotere) 23

9. CORRELATIVE/SPECIAL STUDIES 25

9.1 Plasma Samples (Optional) 25

9.2 Serum Samples 28

10. STUDY CALENDAR 29

11. DATA REPORTING / REGULATORY REQUIREMENTS 32

11.1 Data Management 32

11.2 Safety Meetings 32

11.3 Monitoring 32

12. STATISTICAL CONSIDERATIONS 33

12.1 Study Design 33

12.2 Sample Size/Accrual Rate 34

12.3 Monitoring Plan 36

12.4 Analysis Plans 37

12.5 Expected accrual rate, accrual duration, and total study duration 39

REFERENCES 40

APPENDIX A: Performance Status Criteria 42

APPENDIX B: FACT-P Questionnaire (Version 4) 43

APPENDIX C: Serious Adverse Event Reporting Form 44

1. OBJECTIVES

1.1 Primary Objectives

1. To compare the proportion of metastatic prostate cancer patients with a PSA decline of ≥50% over 8 cycles of docetaxel with ascorbic acid (Arm A) versus docetaxel with placebo (Arm B).

2. To compare the proportion of adverse events (fatigue, nausea, bone pain, and anorexia) experienced by metastatic prostate cancer patients receiving either docetaxel with ascorbic acid (Arm A) versus docetaxel with placebo (Arm B).

1.2 Secondary Objectives

1. To assess radiographic progression free survival (rPFS) in patients with metastatic prostate cancer and compare between treatment arms.

2. To assess the proportion of high grade serious adverse events (fatigue, nausea, bone pain, and anorexia) in patients with metastatic prostate cancer and compare between treatment arms during 8 cycles of treatment.

3. To assess the proportion of high grade serious adverse events (all types) in patients with metastatic prostate cancer and compare between treatment arms during 8 cycles of treatment.

4. To assess changes in quality of life measures as assessed by the FACT-P questionnaire.

5. To assess the proportion of metastatic prostate cancer patients requiring docetaxel dose reductions and compare between treatment arms during 8 cycles of treatment.

6. To assess overall survival (OS) in patients with metastatic prostate cancer and compare between treatment arms.

1.3 Exploratory Objectives

1. To determine whether ascorbic acid alters docetaxel exposure and compare between treatment arms. To correlate docetaxel exposure with clinical outcomes.

2. To determine peak and trough ascorbic acid levels

3. As a pharmacodynamic measure of oxidant injury in vivo, measure F_2-isoprostanes

1.4 Study Design

This is a double-blinded, placebo-controlled, randomized, phase 2 trial of docetaxel with and without I.V. ascorbic acid with the co-primary endpoints of reducing quality-of-life-related-toxicity (fatigue, anorexia, etc.) and improved PSA response. As a secondary endpoint we will monitor radiographic progression free survival, all adverse events, and other measures of quality of life. Sixty-three chemo-naïve men with metastatic castration resistant prostate cancer will be randomized 2:1 to docetaxel with ascorbic acid (Arm A, n=42) or docetaxel with placebo (Arm B, n=21). To allow for 10% drop out rate and 63 evaluable patients, we will accrue up to 69 patients.

The study will consist of a screening period (within 28 days of first dose, inclusive of a randomization period of up to 7 days of first dose), a treatment period (8 Cycles total), and a follow-up period. Dose reductions are allowed per **Section 6**. Enrollment will continue until 63 subjects have received at least one dose of study treatment. Docetaxel will be provided as standard of care while ascorbic acid will be provided by the trial sponsor. Blinding will be utilized in this study to minimize bias. At randomization, the assignment of ascorbic acid versus placebo will be blinded to the patients and the investigator.

All subjects may continue in the treatment period until discontinuation due to unacceptable toxicity, lack of clinical benefit as determined by the investigator, or termination of the study by the sponsor. After a patient is discontinued from treatment, a mandatory Off-Study/Safety Follow-Up Visit should be performed approximately 30 days after the last dose of study medication (or within seven days prior to initiation of a new anti-cancer treatment, whichever comes first). Subjects will be considered in the treatment period until 30 days after the last dose of study drug.

All patients who discontinue treatment should be contacted every six months to monitor overall survival for 3 years or until study closure. Information of other cancer therapies after discontinuation from the study treatment will be collected.

2. BACKGROUND

2.1 Prostate cancer

Prostate cancer (PC) is the most commonly diagnosed malignancy and the second leading cause of cancer death in men. Radical prostatectomy and radiation therapy are the established definitive treatments for clinically localized PC; yet, over 50,000 men each year progress with a biochemical relapse while others develop metastatic disease resulting in over 30,000 deaths annually. While chemical castration is temporarily effective in many patients, most men eventually progress to castrate-resistant prostate cancer (CRPC) and are then treated with docetaxel-based chemotherapy.

2.2 Docetaxel (Taxotere) in Prostate Cancer

The safety and efficacy of docetaxel in combination with prednisone in patients with androgen independent (hormone refractory) metastatic prostate cancer were evaluated in a randomized multicenter active control trial[1]. A total of 1006 patients with Karnofsky Performance Status (KPS) ≥60 were randomized to the following treatment groups:

- Docetaxel 75 mg/m^2 every 3 weeks for 10 cycles.
- Docetaxel 30 mg/m^2 administered weekly for the first 5 weeks in a 6-week cycle for 5 cycles.
- Mitoxantrone 12 mg/m^2 every 3 weeks for 10 cycles

	TAXOTERE+ Prednisone every 3 weeks	Mitoxantrone+ Prednisone every 3 weeks
Number of patients	335	337
Median survival (months)	18.9	16.5
95% CI	(17.0-21.2)	(14.4-18.6)
Hazard ratio	0.761	--
95% CI	(0.619-0.936)	--
p-value*	0.0094	--

*Stratified log rank test. Threshold for statistical significance = 0.0175 because of 3 arms.

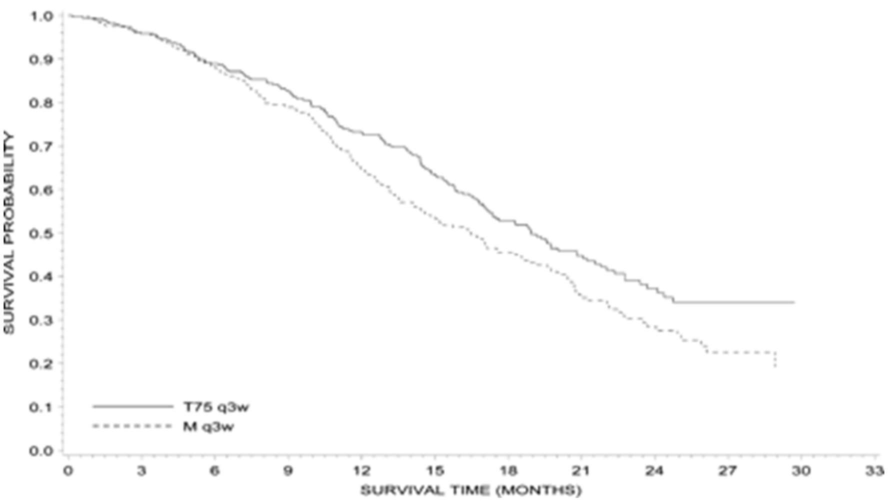

2.3 Clinical Efficacy and Safety of I.V. Ascorbic Acid In Cancer Patients

Multiple phase I trials of IV ascorbic acid safety and phase I/II trials of IV ascorbic acid safety and efficacy in cancer patients have been published. In one trial ascorbic acid in elderly patients with advanced cancer who had failed all other therapies, 2 patients had unexpectedly stable disease after 8 weeks of ascorbic acid treatment [2]. In a trial of patients with metastatic stage 4 pancreatic cancer who received primary therapy with ascorbic acid, gemcitabine, and erlotinib, 8 of 9 patients had tumor shrinkage after 8 weeks of therapy as measured by CT imaging [3]. A phase I trial in patients with metastatic stage 4 pancreatic cancer who were treated with gemcitabine + IV ascorbic acid as primary therapy until tumor progression showed few toxicities associated with the treatment. The 9 patients had a tripling of disease free interval in comparison to literature controls, and doubling of survival compared to retrospective controls [4]. In a dose escalation study, found no toxicity at doses even above 1.5g/kg, the doses used in the other three trials [5]. In addition, a phase I/II trial of high-dose, IV ascorbic acid reduced grade 1 and 2 toxicities associated with carboplatin and paclitaxel treatment by more than 50% in women with ovarian cancer in a small, randomized clinical trial. Patients receiving IV ascorbic acid reported lower levels of low-grade gastrointestinal, hepatobiliary, dermatological, immune/infection, pulmonary and renal toxicities commonly associated with carboplatin and paclitaxel treatment [6]. A trial enrolling women with breast cancer randomized to IV ascorbic acid or placebo, in the first year following surgery, showed significant reductions in nausea (p=0.022), loss of appetite (p=0.005), fatigue (p=0.023), dizziness (p=0.004) and hemorrhagic diathesis (p=0.032). Reduced toxicities were experienced by patients independent of whether they were being treated with chemotherapy [7]. Similarly, a phase I/II study of arsenic trioxide/bortezomib/ascorbic acid combination therapy for the treatment of

relapsed or refractory multiple myeloma with ascorbic acid IV on days 1, 4, 8, and 11 demonstrated a response rate of 27% in a heavily pretreated population. Ascorbic acid demonstrates activity at twice a week dosing.[22]

Intravenous ascorbic acid is in wide use by practitioners of complementary and alternative medicine. Estimates are that in the US approximately 10,000 patients receive IV ascorbic acid each year at average doses of 0.5/kg, with average number of doses of twenty per patient. Using multiple tracking mechanisms, minimal adverse events were reported in properly screened patients [8].

2.4 Ascorbic Acid Mechanism of Action in Prostate Cancer

The mechanism of action of ascorbic acid-mediated tumor suppression appears to depend on hydrogen peroxide production in extracellular fluid; hydrogen peroxide induces necrosis in tumor cells but not in normal cells.[9] Ascorbic acid treatment inhibits prostate cancer cell growth through three mechanisms: (1) ascorbic acid induces H_2O_2-dependent cytotoxicity in prostate cancer cells, (2) ascorbic acid treatment decreased ATP levels in prostate cancer cells, and (3) ascorbic acid treatment induced autophagy in prostate cancer cells.[10] Ascorbic acid treatment also suppresses metastases in hormone-refractory prostate cancer.[11] Cytotoxicity of ascorbic acid requires mM concentrations[12]. The dose response curve for ascorbic acid on DU-15 and PC-3 prostate cancer cells, shown below (Source: Levine, NCI Developmental Therapeutics Program NSC 33832) demonstrate that ascorbic acid was more effective for DU 145, with nearly complete kill at 10 mM, and produced no growth for PC3.

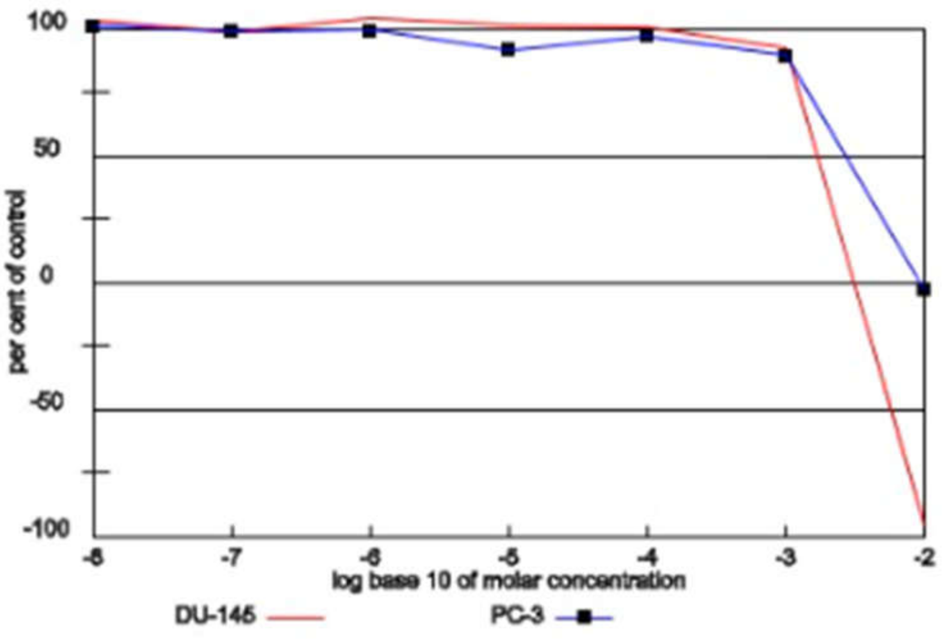

Bioavailability of ascorbic acid is tightly controlled in response to oral administration and reaches only 100 μM with consumption of five servings of fruits and vegetables, which contain approximately 250 mg of ascorbic acid. Oral supplementation even at many grams does not increase plasma concentrations beyond 250 μM. IV administration bypasses the tight controls found with oral administration, and produces pharmacological ascorbic acid concentrations of 25-30 mM [13]. Pharmacological ascorbic acid concentrations of 10mM can be maintained for approximately 4 hours with infusion rates of 0.5 – 1.0 g/kg per minute [2,9].

2.5 Rationale for Combination Therapy With Ascorbic Acid and Chemotherapy

Additivity/Synergy: The combination of ascorbic acid and conventional chemotherapeutic agents synergistically inhibited ovarian cancer in mouse models. An additive to synergistic effect was shown for ascorbic acid and carboplatin in OVCAR5 and SHIN3 cells at all combination ratios, and in OVCAR8 cells at a high ascorbic acid ratio. Epithelial cell HIO-80 was sensitive to carboplatin treatment but the addition of ascorbic acid induced no more cell death than carboplatin alone.[6] Clinically, several trials have demonstrated safety of ascorbic acid in combination with chemotherapy, including in ovarian cancer, multiple myeloma, and pancreatic cancer. [3, 4, 6, 22]

Drug-Drug Interaction Potential: A drug-drug interaction between ascorbic acid and docetaxel is not anticipated based on drug metabolism. Docetaxel metabolism is primarily mediated by CYP3A4/5 and to a much lesser extent CYP2C8 and CYP1B1. Clinical drug-drug interactions have been noted with strong CYP3A4 inducers and inhibitors. Docetaxel disposition is mediated by the following drug transporters: ABCB1, ABCC1, ABCC2, ABCG2, SLCO1A2, SLCO1B1, and SLCO1B3[14]. Additionally, docetaxel is bound to alpha1-acid glycoprotein levels (AAG), which is a stress-reactive protein and has been linked with docetaxel exposure[15]. It is not anticipated that ascorbic acid alters any of these processes. Therefore, formal docetaxel pharmacokinetics will not be collected except in patients willing to undergo optional PK. For those patients, sparse sampling for docetaxel exposure data will be collected in Cycle 1 (docetaxel in combination with ascorbic acid administration). In addition, sparse sampling for docetaxel exposure data will be collected if adverse drug events are more frequent and severe as deemed by the treating oncologist and/or principal investigator of the trial. The collection would occur in the subsequent cycle (C2 and beyond) from when the adverse drug events occur.

2.6 Justification for Using IV Ascorbic Acid with Docetaxel Prostate Cancer at Johns Hopkins

Many men with late stage prostate cancer are treated with docetaxel and experience significant toxicities including fatigue, alopecia, diarrhea, stomatitis, and peripheral edema[16]. Given the efficacy of IV ascorbic acid in reducing toxicities in other cancers, a rigorous evaluation of IV ascorbic acid in reducing toxicities associated with docetaxel treatment in men with metastatic prostate cancer is merited.

The Sidney Kimmel Comprehensive Cancer Center at Johns Hopkins has a large population of men with metastatic castration resistant prostate cancer who are treated with docetaxel.

We propose to conduct a randomized, controlled phase 2 trial of docetaxel with and without IV ascorbic acid with the primary endpoint of reducing toxicity. A secondary endpoint, progression free survival will be measured to determine whether the anti-cancer effects of IV ascorbic acid are sufficient to merit larger trials. We also plan to measure correlates, including ascorbic acid levels in blood and F_2-isoprostanes levels indicative of oxidative stress[4], to help shed light on the mechanism by which IV ascorbic acid reduces toxicities.

3. PATIENT SELECTION

3.1 Eligibility Criteria

Patients are eligible to be included in the study only if they meet ***all*** of the following criteria:

[1] Have metastatic castration-resistant prostate cancer (prostate cancer progressing despite castrate levels of testosterone [< 50 ng/dL] using standard measures of progression defined by Prostate Cancer Working Group 2[17]), are chemo-naïve for mCRPC. Patients must have symptomatic disease or visceral metastases or otherwise be eligible for docetaxel treatment per investigator judgment (e.g. for progression on imaging or rapidly rising PSA despite 2nd line hormonal treatment).

Note: Six cycles of prior docetaxel are allowed in hormone-sensitive disease (per ECOG 3805 data) and have been off of docetaxel for at least 12 months.

[2] Have a pathological diagnosis of prostate carcinoma

[3] Patients may be receiving continuous hormonal ablation with surgical or medical castration with baseline testosterone <50ng/dL

[4] Patient may be receiving bone targeted agents.

[5] Have evaluable disease by RECIST 1.1 and/or PCWG2 criteria.

[6] Age ≥ 18.

[7] Have ECOG performance status 0-1 (**Appendix A**).

[8] Have estimated life expectancy > 4 months.

[9] Have normal organ and marrow function as defined below:

- Absolute neutrophil count	≥ 1500/mm³
- Platelets	≥ 100,000/mm³
- Hemoglobin	≥ 9 g/dL
- Total bilirubin	≤ 1.0 ULN
- AST(SGOT)/ALT(SGPT)	≤ 2.5 x ULN
- Creatinine	≤ 1.6 mg/dl (for patients with >1.6 mg/dl, Calculated creatinine clearance must be ≥ 60 mL/minute (Cockcroft-Gault)).

[10] Men of reproductive potential and those who are surgically sterilized (i.e., post vasectomy) must agree to practice effective barrier contraception that has an expected failure rate of < 1% during and for 30 days after discontinuation of study treatment.

- If condoms are used as a barrier contraceptive, a spermicidal agent should be added to ensure that pregnancy does not occur.

[11] Have the ability to understand, and have given written informed consent before performance of any study-related procedure not part of normal medical care, with the understanding that consent may be withdrawn by the subject at any time without prejudice to future medical care.

3.2 Exclusion Criteria

Patients are ineligible to be included in the study if they:

[1] Have had known active central nervous system (CNS) metastases and/or carcinomatous meningitis. Subjects with previously treated brain metastases may participate provided they are stable (without evidence of progression by imaging for at least four weeks prior to the first dose of trial treatment and any neurologic symptoms have returned to baseline), have no evidence of new or enlarging brain metastases, and are not using steroids for CNS involvement for at least one week prior to trial treatment. Patients with primary brain tumors are not eligible. However, as patients are completing abiraterone therapy, they will be allowed to continue up to 10 mg/day of prednisone.

[2] Have had prior chemotherapy for metastatic disease in castration-resistant prostate cancer (prior chemotherapy for hormone-sensitive disease, more than twelve months prior to registration, is acceptable).

[3] Have had had surgery within four weeks of dosing of investigational agent, excluding minor procedures (dental work, skin biopsy, etc.), celiac plexus block, and biliary stent placement.

[4] Have had palliative radiation or biological cancer therapy within 2 weeks prior to the first dose of study drug.

[5] Have received other investigational drugs within 28 days prior to enrollment.

[6] Is expected to require any other form of systemic or localized antineoplastic therapy while on study.

[7] Patients who require frequent (several times a day) monitoring of their blood glucose or patients who have recently been hospitalized for glucose control.

[8] Are being treated with warfarin anticoagulation therapy.

[9] The subject requires concomitant treatment with the following inhibitors of CYP3A4:
- Antibiotics: clarithromycin, erythromycin, telithromycin, troleandomycin
- Antifungals: itraconzaole, ketoconazole, voriconazole, fluconazole, posaconazole
- Antidepressants: nefazodone
- Antidiuretic: conivaptan
- Antiretrovirals: delaviridine or protease inhibitors (ritonavir, indinavir, lopinavir/ritonavir, saquinavir, nelfinavir) or cobicistat-boosted antiretrovirals.
- GI: cimetidine, aprepitant
- Hepatitis C: boceprevir, telaprevir

- Miscellaneous: Seville oranges, grapefruit, or grapefruit juice and/or pummelos, star fruit, exotic citrus fruits, or grapefruit hybrids).

Because the lists of CYP3A4 inhibitors are constantly changing, it is important to regularly consult a frequently-updated list such as **http://medicine.iupui.edu/clinpharm/ddis/table.aspx**; medical reference texts such as the Physicians' Desk Reference may also provide this information. As part of the enrollment/informed consent procedures, the patient will be counseled on the risk of interactions with other agents, and what to do if new medications need to be prescribed or if the patient is considering a new over-the-counter medicine or herbal product

[10] Have uncontrolled intercurrent illness, including but not limited to ongoing or active infection, symptomatic congestive heart failure, unstable angina pectoris, cardiac arrhythmia, or psychiatric illness/social situations that would limit compliance with study requirements.

[11] Has glucose-6-phosphate dehydrogenase (G6PD) deficiency (Red blood cell hemolysis may occur in people found to be deficient in the G6PD enzyme).

[12] Have end stage renal disease

[13] Has history of calcium oxalate stones

[14] Has history of iron overload

[15] Have a known history of Human Immunodeficiency Virus (HIV) (HIV 1/2 antibodies).

[16] Have a known active uncontrolled hepatitis B, or hepatitis C infection.

4. REGISTRATION PROCEDURES

4.1 General Guidelines

Eligible patients will be entered on study centrally at the Sidney Kimmel Comprehensive Cancer Center at the Johns Hopkins University by the Lead Study Coordinator. All sites should call/email the coordinating center at crocc@jhmi.edu to verify drug availabilities. The Registration Form, and Eligibility Worksheet will be supplied to each participating site.

If a patient does not receive protocol therapy following registration, the patient's registration on the study may be canceled. The Coordinating Center should be notified of cancellations as soon as possible.

4.2 Registration Process

To register a patient, the following documents should be completed by the Research Nurse or Study Coordinator and emailed to crocc@jhmi.edu and Amber Michalik to the Coordinating Center:

- Registration Form
- Signed patient consent form
- HIPAA authorization form
- Eligibility Screening Checklist
- Copy of required screening tests and scans

The Research Nurse or Study Coordinator at the participating site will then e-mail (crocc@jhmi.edu and amichal2@jhmi.edu) the Coordinating Center (Amber Michalik) to verify eligibility. To complete the registration process, the Coordinating Center will:

- Assign a patient study number
- Register the patient on the treatment portion of the study with the Sidney Kimmel Comprehensive Cancer Center's Clinical Research Office
- Fax or e-mail the patient study number to the participating site
- Call or e-mail the research nurse or data manager at the participating site and verbally confirm registration the last eligible start date for treatment.

4.3 Multicenter Guidelines

Protocol Chair

The Protocol Chair is responsible for performing the following tasks:

- Coordinating, developing, submitting, and obtaining approval for the protocol as well as its subsequent amendments
- Assuring that all participating institutions are using the correct version of the protocol.
- Taking responsibility for the overall conduct of the study at all participating institutions and for monitoring the progress of the study.
- Reviewing and ensuring reporting of Serious Adverse Events (SAE)
- Reviewing data from all sites

Coordinating Center

The Coordinating Center is responsible for performing the following tasks:

- Ensuring that IRB approval has been obtained at each participating site prior to the first patient registration at that site, and maintaining copies of IRB approvals from each site.
- Managing central patient registration.
- Collecting and compiling data from each site.
- Establishing procedures for documentation, reporting, and submitting of AE's and SAE's to the Protocol Chair, and all applicable parties.
- Facilitating audits by securing selected source documents and research records from participating sites for audit, or by auditing at participating sites.

Participating Sites

Participating sites are responsible for performing the following tasks:

- Following the protocol as written, and the guidelines of Good Clinical Practice (GCP).
- Submitting data to the Coordinating Center.
- Registering all patients with the Coordinating Center by submitting patient registration form, and signed informed consent promptly.

- Providing sufficient experienced clinical and administrative staff and adequate facilities and equipment to conduct a collaborative trial according to the protocol.
- Maintaining regulatory binders on site and providing copies of all required documents to the Coordinating Center.
- Collecting and submitting data according to the schedule specified by the protocol.

5. TREATMENT PLAN

5.1 Agent Administration

Treatment will be administered on an outpatient basis. Reported adverse events and potential risks are described in **Section 7**. Appropriate dose modifications are described in **Section 6**. No investigational or commercial agents or therapies other than those described below may be administered with the intent to treat the patient's malignancy. At randomization, the assignment of ascorbic acid versus placebo will be blinded to the patients and investigators.

REGIMEN DESCRIPTION					
**Agent**	**Premedications; Precautions**	**Dose**	**Route***	**Schedule**	**Cycle Length**
**Docetaxel**	Decadron 8 mg po 12 hours, 3 hours and 1 hour prior to docetaxel administration, ; 5-HT3 antagonist IV on Day 1, 5-HT3 antagonist PRN. Prednisone 5mg po BID continuously	75 mg/m^2	IV over 60 min*	Q 3 weeks (Day 1 of each cycle)	21 days (3 weeks) for a total of 8 cycles
**Ascorbic acid**	None	1g/kg** (For first cycle and for first cycle in the extension phase only: titrate first 3 doses: 0.25, 0.5, and 0.75g/kg) in 1000ml of water (Not to exceed 500 mL per hour.)	0.75-1 gram per minute IV*	2 times per week*** First dose on day 1 of the cycle	
**Placebo (Normal Saline)**	None	1000ml of normal saline	0.75-1 gram per minute IV*	2 times per week***First dose on day 1 of the cycle	

*Infusion times are approximate (± 15 minutes) and may need to be adjusted based on patient tolerability. Infusion time is estimated to be between 90-120 minutes.
**For a subject weighing 75 kg, the goal dose is 75 grams (1 g/kg), then the titrated dose schedule is 18.75 g, 37.5 g and 56.25g for cycle 1 only. Rounding the calculated doses to 19 g, 38 g and 56 g is acceptable. Subsequent doses would be 75 g. The maximum allowed dose for patients weighing over 100 kg is 100g.
***The ideal schedule for ascorbic acid is 3-4 days apart for administration. However, ascorbate can be safely administered on consecutive days (at least 24 hours apart). The day that ascorbate or placebo can be administered can be shifted in the event of holidays, inclement weather, and other reasons for clinic closure to even three consecutive days.

Subsequent ascorbic acid infusions may not be given less than 24 hours apart.

Acute reactions will be managed using standard therapy for acute drug reactions as per institutional standard of care.

5.2 Dosing Criteria

To continue therapy, subjects must meet the following criteria (exceptions should be approved by the principal investigator):
For Docetaxel:

- Absolute Neutrophil Count ≥1000/mcL
- Platelets ≥80 x 10^9/L
- Hemoglobin > 8 g/dL
- Total bilirubin ≤ 1.0 ULN
  AST(SGOT)/ALT(SGPT) ≤ 2.5 x ULN

For Ascorbic acid: Creatinine ≤ 1.6 mg/dl (for patients with ≥1.6 mg/dl, Calculated creatinine clearance must be ≥ 60 mL/minute (Cockcroft-Gault)).

5.3 General Concomitant Medication and Supportive Care Guidelines

The concurrent use of all other drugs, over-the-counter medications, or alternative therapies must be documented. Supportive care, including but not limited to anti-emetic medications, may be administered at the discretion of the Principal Investigator or Co-Investigators. Concurrent treatment with bisphosphonates is allowed. All concomitant treatments, including blood and blood products, must be reported on the case report form (CRF). Erythropoietin, G-CSF, or Pegfilgrastim may be administered at the discretion of the Principal Investigator or Co-Investigators. Warfarin anticoagulant therapy is not allowed[18,19]. Heparin, enoxaparin, aspirin, NSAIDS, or other anticoagulant therapies are allowed.

5.4 Expected Adverse Events

5.4.1 Ascorbic Acid

Too rapid intravenous administration of the solution may cause temporary faintness or dizziness.

The most common side effects of ascorbic acid include: nausea, vomiting, diarrhea, dry mouth, loss of appetite, muscle weakness, dizziness, headache, fatigue, flushing, leakage of protein, injection site reaction (including transient pain, swelling at the site), fever, chills.

Less common side effects of ascorbic acid include: kidney stone, renal failure, hypernatremia, hypoalbuminemia, hyperglycemia, hypokalemia increase in blood pressure, severe headache, sclerosing at the injection site and phlebitis.

Rare side effects of Ascorbic Acid include: respiratory and cutaneous allergies to Ascorbic Acid

Of note, it has been reported that patients with a history of glucose 6 phosphate dehydrogenase deficiency experienced hemolysis.

Deep-vein thrombosis has been reported after large doses of ascorbic acid.

Rarely, decreased blood pH leading to sickle-cell crisis has been reported in patients with sickle cell disease.

At doses of greater than 600 mg, ascorbic acid has been reported to have a diuretic action.

High doses can increase serum cholesterol in atherosclerotic patients.

5.4.2 Docetaxel

Most common adverse reactions across all docetaxel indications are infections, neutropenia, anemia, febrile neutropenia, hypersensitivity, thrombocytopenia, neuropathy, dysgeusia, dyspnea, constipation, anorexia, nail disorders, fluid retention, asthenia, pain, nausea, diarrhea, vomiting, mucositis, alopecia, skin reactions, myalgia.

Other potential adverse reactions include: epistaxis, allergic reactions, rash/desquamation, stomatitis/pharyngitis, cough, dyspnea, cardiac left ventricular function, fatigue, tearing, arthralgia, and kidney failure.

5.5 Duration of Therapy and Criteria for Removal from Study

Patients will be removed from study when any of the criteria listed in apply. The reason for study removal and the date the patient was removed must be documented in the Case Report Form.

In the absence of treatment delays due to adverse event(s), treatment may continue for 8 cycles or until one of the following criteria applies:

- Disease progression by RECIST or bone progression per PCWG2 (see below),
- Intercurrent illness that prevents further administration of treatment,

- Unacceptable adverse event(s): toxicity (defined in **Section 6.1**) that has not recovered in 2 weeks or if the same toxicity recurs at the decreased dose (unless there is strong evidence of clinical benefit to justify continuation of dosing with study treatment, and the investigator must discuss the rationale with the Sponsor before a decision is taken),

- ≥ Grade 3 hypersensitivity to docetaxel or ascorbic acid

- Patient decides to withdraw from the study, or

- General or specific changes in the patient's condition render the patient unacceptable for further treatment in the judgment of the investigator.

An Open Label Extension Phase will be permitted to those patients that have completed the study treatment (cycles 1-8), or for patients removed due to protocol defined progression by RECIST. The decision to continue treatment beyond initial progression must be documented in the study records. Patients can continue on docetaxel standard of care and ascorbic acid or ascorbic acid alone. Ascorbic acid will be available twice a week until progression. Patients will be required to get safety labs (CBC and CMP weekly for ascorbic acid), and be assessed for progression per investigator judgment and at least every 12 weeks by PSA, CMP, CBC, bone scan, CT scan, and H&P. The History & Physical can be performed on Day 1 of each cycle during the Open Label Extension Phase (as it is during cycles 1-8), however it is only required to be performed every 12 weeks, unless clinically indicated and at the investigators discretion. Research bloods and the FACT-P Questionnaire will not be collected during the Open Label Extension Phase.

When subjects come off treatment in the Open Label Extension Phase and move to Follow-up, the serum samples will need to be collected during the 30 Day Follow-up visit. A CT scan and bone scan should also be completed at the 30 Day Follow-up visit unless the scans have already been completed within the past 6 weeks. The site study team must inform the site pharmacy when subjects will be switching over to the Open Label Extension Phase.

Criteria for Removal from Study

Disease Progression
Progression will be evaluated on the basis of biochemical, radiographic, or symptomatic progression based on modified PCWG2 criteria. Determinations of disease progression will be made by the investigator.

Progression will be defined as follows:

1. Radiographic progression defined by at least one of the following:
   - Soft tissue disease progression by modified RECIST criteria v.1.1 (baseline LN size must be ≥ 2.0 cm to be considered target or evaluable lesion)
   - Development of two or more new bone lesions not consistent with tumor flare per PCWG2.
2. Symptomatic or clinical progression defined by one of the following:
   - Development of a skeletal related event (SRE) defined as pathologic fracture, spinal cord or nerve root compression, palliative radiation to bone, or surgery to bone
   - Worsening pain due to CRPC unable to be controlled by non-narcotic or narcotic medications

- Sustained worsening of ECOG status resulting from CRPC (e.g., 2 to 3)
- Treating physician decides to initiate new systemic anti-cancer therapy

We will encourage investigators to continue study treatment until radiographically confirmed disease progression requires initiation of new systemic antineoplastic therapy. In addition we will measure PSA levels but do not plan to use PSA progression as a reason for cessation of treatment.

5.6 Duration of Follow Up

Subjects will be followed for adverse events for a minimum of 30 days after the last dose of study drug or death, whichever occurs first. Survival status will be collected every 6 months (+/- 4 weeks) by phone or chart review for 3 years or until study closure. Subsequent therapies and responses may be collected. Subjects removed from study for unacceptable adverse event(s) will be followed until resolution or stabilization of the adverse event or death.

6. DOSING DELAYS/DOSE MODIFICATIONS

6.1 Dosing Delays

All scheduled cycles occur over a 21-day period. If necessary, treatment may be delayed for up to two weeks. If treatment is delayed more than two weeks, the Sponsor must be contacted for further instructions on continued treatment. Additional delays or modifications to the treatment schedule must be approved by the IND Sponsor. When the delay is due to toxicity, then the next cycle may begin with a dose reduction. If docetaxel needs to be delayed for any reason, ascorbic acid/placebo will also need to be delayed and started back up at the same time as docetaxel.

Docetaxel will be withheld for the following drug-related toxicities:

- Absolute neutrophil count (ANC) <500/uL for longer than seven days
- ANC <1000/uL with a single temperature of >101ºF/38.3ºC or a sustained temperature of >100.4ºF/38ºC for more than one hour
- Platelet count < 80 x 10^3/uL
- Hemoglobin < 8 g/dL
- Common Terminology Criteria for Adverse Events (CTCAE) Grade 3 or 4 non-hematologic toxicity (for example, including gastrointestinal toxicity) excluding asymptomatic electrolyte aberrations.

Any AE, laboratory abnormality, or intercurrent illness that, in the judgment of the investigator, warrants delaying the dose of study medication. Ascorbic acid will be withheld for the following drug-related toxicities:

- Creatinine > 1.6 mg/dl (for patients with >1.6 mg/dl, calculated creatinine clearance must be < 60 mL/minute (Cockcroft-Gault)).

- Common Terminology Criteria for Adverse Events (CTCAE) Grade 3 or 4 non-hematologic toxicity (for example, including gastrointestinal toxicity) excluding asymptomatic electrolyte aberrations.

- Any AE, laboratory abnormality, or intercurrent illness that, in the judgment of the investigator, warrants delaying the dose of study medication

Management of gastrointestinal toxicity:

**Event Name**	**Nausea**
**Grade of Event**	**Management/Next Dose**
Grade 1	No change in dose
Grade 2	No change in dose
Grade 3-4	Hold until ≤ Grade 2. Resume at one dose level lower, if indicated per **Section 6.2**.
Recommended management: antiemetics.	

**Event Name**	**Vomiting**
**Grade of Event**	**Management/Next Dose**
Grade 1	No change in dose
Grade 2	No change in dose
Grade 3-4	Hold until ≤ Grade 2. Resume at one dose level lower, if indicated per **Section 6.2**.
Recommended management: antiemetics.	

**Event Name**	**Diarrhea**
**Grade of Event**	**Management/Next Dose**
Grade 1	No change in dose
Grade 2	No change in dose
Grade 3-4	Hold until ≤ Grade 2. Resume at one dose level lower, if indicated per **Section 6.2**.
Recommended management: Loperamide anti-diarrheal therapy<br>Dosage schedule: 4 mg at first onset, followed by 2 mg with each loose motion until diarrhea-free for 12 hours (maximum dosage: 16 mg/24 hours)<br>Adjunct anti-diarrheal therapy is permitted and should be recorded when used.	

Management of peripheral neuropathy:

**Event Name**	**Peripheral Neuropathy**
**Grade of Event**	**Management/Next Dose**
Grade 1	No change in dose
Grade 2	Resume at one dose level lower if symptoms interfering with instrumental ADL
Grade 3-4	Discontinue docetaxel
Recommended management: consider medications for neuropathy.	

In case toxicity does not resolve to Grade 0-2 within two weeks after the last dose (except creatinine that must resolve to dosing criteria listed in **Section 5.2**), trial treatment should be discontinued after consultation with the Sponsor (with the exception of alopecia, fatigue, skin rash, nausea, vomiting, constipation, or diarrhea that can be controlled with treatment) unless there is strong evidence of clinical benefit to justify continuation of dosing with study treatment, and the investigator must discuss the rationale with the Sponsor before a decision is taken.

Altering the ascorbate/placebo or docetaxel schedule due to scheduling and holidays is permitted. For ascorbate/placebo, it is preferred that 2 doses are administered per week. Ascorbic acid infusions may not be given less than 24 hours apart. If warranted, the correlative study assessments for ascorbic acid should be adjusted to the adjusted infusion days.

6.2 Dose Modifications

Patients who experience grade 3 or 4 toxicities (individually defined in **Section 6.1**) that are eligible for retreatment will be dose reduced. After a two-week dose delay, if the grade 3 or 4 toxicity resolves to grades 0-2 (grade 2 peripheral neuropathy must resolve to grade 1 if interfering with instrumental ADL), then the following dose reductions are to be applied:

- One dose modification of ascorbic acid is allowed to 0.75g/kg (if two dose reductions are required, the patient will go off protocol treatment).

- Two dose modifications of docetaxel are allowed to 60 mg/m^2 and 50 mg/m^2 (if three dose reductions are required, the patient may go off protocol treatment at the IND Sponsor's discretion).

Dose reductions for either drug for symptoms other than grade 3 or 4 toxicities should be discussed with and approved by the IND sponsor.

7. ADVERSE EVENTS: LIST AND REPORTING REQUIREMENTS

This study will use the descriptions and grading scales found in the revised CTCAE version 4.0 for AE reporting that can be found at http://ctep.cancer.gov/protocolDevelopment/electronic_applications/ctc.htm.

Information about all AEs, whether volunteered by the subject, discovered by investigator questioning, or detected through physical examination, laboratory test or other means, will be collected, recorded, and followed up as appropriate.

All AEs and SAEs experienced by subjects will be collected and reported from the first dose of the investigational agent, throughout the study, and will only be followed for four weeks unless related to the investigational agent. All SAEs will be collected for four weeks after the last dose of the investigational agent. Although this responsibility is usually shared among the PI, research nurse, and data manager, the PI is ultimately responsible for grading and attribution of all events.

Subjects who have an ongoing AE related to the study procedures and/or medication(s) may continue to be periodically contacted by a member of the study staff until the event is resolved or determined to be irreversible by the investigator.

Laboratory abnormalities: Laboratory abnormalities present at the screening visit will be recorded as pre-treatment signs and symptoms. After study treatment administration, all Grade 3 and 4 clinical laboratory results that represent an increase in severity from baseline will be reported as AEs. A Grade 1 or 2 clinical laboratory abnormality should be reported as an AE only if it is considered clinically significant by the investigator.

7.1 Definitions

7.1.1 Adverse Event

An AE is defined as any undesirable sign, symptom, or medical condition occurring after starting the study drug (or therapy), even if the event is not considered to be related to the study. An undesirable medical condition can be symptoms (e.g., nausea, chest pain), signs (e.g., tachycardia, enlarged liver), or the abnormal results of an investigation (e.g., laboratory findings, electrocardiogram). Medical conditions/diseases present before starting the study treatment are only considered AEs if they worsen after starting the study treatment (any procedures specified in the protocol). AEs occurring before starting the study treatment but after signing the informed consent form will not be recorded. Abnormal laboratory values or test results constitute AEs only if they induce clinical signs or symptoms or require therapy.

7.1.2 Serious Adverse Event

A SAE is an undesirable sign, symptom, or medical condition that:

- Results in death
- Is life threatening (defined as an event in which the subject was at risk of death at the time of the event; it does not refer to an event that hypothetically might have caused death if it were more severe)
- Requires inpatient hospitalization or causes prolongation of existing hospitalization (see note below for exceptions) for >24 hours
- Results in persistent or significant disability/incapacity
- Is a congenital anomaly/birth defect (note: reports of congenital anomalies/birth defects must also be reported on the Pregnancy Supplemental Form)
- Is an important medical event (defined as a medical event(s) that may not be immediately life-threatening or result in death or hospitalization but, based upon appropriate medical and scientific judgment, may jeopardize the subject or may require intervention [e.g., medical, surgical] to prevent one of the other serious outcomes listed in the definition above). Examples of such events include but are not limited to intensive treatment in an emergency room or at home for allergic bronchospasm; and blood dyscrasias or convulsions that do not result in hospitalization.
- Is a new cancer (that is not a condition of the study)
- Is associated with an overdose

Events ***not*** considered to be SAEs are hospitalizations for:

- Admissions as per protocol for a planned medical/surgical procedure or to facilitate a procedure
- Routine health assessment requiring admission for baseline/trending of health status (e.g., routine colonoscopy)

- Medical/surgical admission for purpose other than remedying ill health state and that was planned prior to entry into the study. Appropriate documentation is required in these cases.
- Admission encountered for another life circumstance that carries no bearing on health status and requires no medical/surgical intervention (e.g., lack of housing, economic inadequacy, care-giver respite, family circumstances, administrative).
- Admissions for monitoring of treatment-related infusion reactions that do not otherwise meet the criteria for a SAE.

7.2 Relationship

The relationship of an AE to the administration of the study drug is to be assessed by the investigator according to the following definitions:

- No (unrelated, not related, no relation): The time course between the administration of the study drug and the occurrence or worsening of the adverse event rules out a causal relationship, and another cause (concomitant drugs, therapies, complications, etc.) is suspected.

- Yes (related): The time course between the administration of the study drug and the occurrence or worsening of the adverse event is consistent with a causal relationship and no other cause (concomitant drugs, therapies, complications, etc.) can be identified.

The following factors should also be considered:

- The temporal sequence from study drug administration: The event should occur after the study drug is given. The length of time from study drug exposure to event should be evaluated in the clinical context of the event.
- Underlying, concomitant, intercurrent diseases: Each report should be evaluated in the context of the natural history and course of the disease being treated and any other disease the subject may have.
- Concomitant medication: The other medications the subject is taking or the treatment the subject receives should be examined to determine whether any of them might be recognized to cause the event in question.
- Known response pattern for this class of study drug: Clinical and/or preclinical data may indicate whether a particular response is likely to be a class effect.
- Exposure to physical and/or mental stresses: The exposure to stress might induce adverse changes in the recipient and provide a logical and better explanation for the event.
- The pharmacology and pharmacokinetics of the study drug: The known pharmacologic properties (absorption, distribution, metabolism, and excretion) of the study drug should be considered.

7.3 Expectedness

Unexpected adverse event: An AE that varies in nature, intensity, or frequency from information on the investigational drug/agent provided in the product IB, package insert, or safety reports. Any AE that is not included in the IB consent is considered “unexpected.”

Expected (known) adverse event: An AE that has been reported in the IB. An AE is considered “expected” only if it is included in the IB document as a risk.

7.4 Handling of Expedited Safety Reports

In accordance with local regulations, the IND Sponsor will notify investigators of all SAEs that are unexpected (i.e., not previously described in the IB), and related to the investigational agent. This notification will be in the form of an expedited safety report (ESR) that is to be faxed to the investigators and the study coordinators. Upon receiving such notices, the investigator must review and retain the notice with the IB and, where required by local regulations, submit the ESR to the appropriate IRB. The investigator and IRB will determine if the informed consent requires revision. The investigator should also comply with IRB procedures for reporting any other safety information.

7.5 Reporting

7.5.1 General

All AEs (both expected and unexpected) will be captured on the appropriate study-specific CRFs.

In addition, all SAEs, regardless of causality to study drug and/or administration device, will be reported promptly to the IND sponsor (fax: 410-502-9933; e-mail: crocc@jhmi.edu and eprophe1@jhmi.edu), within 24 hours of recognition of the event (**Appendix C**). If this falls on a weekend or holiday, an email notification is acceptable but must be followed by an SAE reporting form on the next business day.

Coordinating Center
The Coordinating Center is the central location for the collection and maintenance of documentation of adverse events and is responsible for submitting adverse event reports to the Protocol Chair promptly. The Coordinating Center will maintain documentation of all adverse event reports for each participating site. Adverse event reports submitted to the Coordinating Center must be signed and dated by the participating site's Principal Investigator. The Coordinating Center will provide appropriate forms to be used by all participating sites for reporting adverse events. Information to be provided must include:

- Subject ID number, and initials
- Date of the event
- Description of the event
- Description of site's response to the event
- Assessment of the subject's condition
- Subject's status on the study (on study, off study, etc.)
- Attribution of event to study drug

Participating Sites
Participating sites are responsible for reporting adverse events to their IRB according to its specific requirements and to the Coordinating Center as follows:

Fatal Events whether anticipated or unanticipated, and whether or not related to the study

must be reported to the Coordinating Center within **24 hours** of the participating site Principal Investigator's learning of the event.

Serious and Unanticipated Adverse Events as defined above must be reported to the Coordinating Center within **24 hours** of the participating site Principal Investigator's learning of the event.

Other Serious Adverse Events which may result in a change to the protocol, informed consent, or risk to subjects as specified in the protocol must be reported within **three (3) working days** of the participating site Principal Investigator's learning of the event.

Adverse Events which result in no change to protocol, informed consent, or risk to subjects must be reported to the Coordinating Center on a monthly basis.

Adverse event reports are to be faxed to the Coordinating Center at SKCCC. Follow-up reports are faxed, mailed, or sent electronically to the Coordinating Center as necessary. The investigator must also report follow-up information about SAEs within the same time frames.

If a non-serious AE becomes serious, this and other relevant follow-up information must also be provided within the same time frames described above.

All SAEs must be collected whether or not they are considered causally related to the investigational product. Investigators and other site personnel are responsible for reporting all casually related SAEs to their IRB and the Protocol Chair.

Although pregnancy and lactation are not considered AEs, it is the responsibility of investigators or their designees to report the pregnancy of a male patient's female partner within 4 weeks days of completing the trial. All pregnancies of female partners of patients must be followed to the completion/termination of the pregnancy. Pregnancy outcomes of spontaneous abortion, missed abortion, benign hydatidiform mole, blighted ovum, fetal death, intrauterine death, miscarriage, and stillbirth must be reported as SAEs (Important Medical Events). If the pregnancy continues to term, the outcome (health of infant) must also be reported to the IND sponsor and Lilly.

7.5.2 Institutional Review Board

All SAEs will be reported to the IRB per institutional standards within three business days of recognition of the AE if the event is related and expected, related and unexpected, or related and fatal or life-threatening due to administration of the investigational product. If the SAE is unrelated to administration of the investigational agents, then it should be reported to the IRB per institutional guidelines. Follow-up information will be submitted to the IRB as soon as relevant information is available.

7.5.3 Food and Drug Administration

All reporting to the Food and Drug Administration (FDA) will be completed by the IND Sponsor.

7.5.3.1 Expedited IND Safety Reports:

Seven Calendar-Day Telephone or Fax Report:
The IND Sponsor is required to notify the FDA of any fatal or life-threatening adverse event that is unexpected and assessed by the investigator to be possibly related to the investigational agent. Such reports are to be telephoned or faxed (301-827-9796) to the FDA within seven calendar days of first learning of the event. Follow-up information will be submitted to the FDA as soon as relevant information is available.

15 Calendar-Day Written Report:
The IND Sponsor is required to notify the FDA of any SAE that is unexpected and related to the investigational agent in a written IND Safety Report.

Written IND Safety Reports should include an Analysis of Similar Events in accordance with regulation 21 CFR § 312.32. All safety reports previously filed with the IND concerning similar events should be analyzed. The new report should contain comments on the significance of the new event in light of the previous related reports.

Written IND safety reports with Analysis of Similar Events are to be submitted to the FDA within 15 calendar days of first learning of the event. Follow-up information will be submitted to the FDA as soon as relevant information is available.

7.5.3.2 IND Annual Reports

In accordance with regulation 21 CFR § 312.33, the IND Sponsor shall within 60 days of the anniversary date that the IND went into effect submit a brief report of the adverse events and progress of the investigation. Please refer to Code of Federal Regulations, 21 CFR § 312.33, for a list of the elements required for the annual report. All IND annual reports will be submitted to the FDA by the IND Sponsor.

8. PHARMACEUTICAL INFORMATION

8.1 Ascorbic acid

8.1.1 Agent Accountability

The sponsor/investigator shall take responsibility for and shall take all steps to maintain appropriate records and ensure appropriate supply, storage, handling, distribution, and usage of investigational products in accordance with the protocol and any applicable laws and regulations.

8.1.2 Mode of Action

Ascorbic Acid (vitamin C) is a water-soluble vitamin. In humans, an exogenous source of ascorbic acid is required for collagen formation and tissue repair. Ascorbic acid is reversibly oxidized to dehydroascorbic acid in the body. These two forms of the vitamin are believed to be important in oxidation-reduction reactions. The vitamin is involved in tyrosine metabolism, conversion of folic acid to folinic acid, carbohydrate metabolism,

synthesis of lipids and proteins, iron metabolism, resistance to infections, and cellular respiration.

8.1.3 Description

Ascor L 500® (Ascorbic Acid Injection, USP) 500mg/mL, is available in a 50-mL sterile dispensing vial. Ascorbic Acid injection is a clear, colorless to slightly yellow sterile, solution of Ascorbic Acid in Water for Injection.

8.1.4 Preparation

When dispensing vials, use aseptic technique. Dispense entire contents in aliquots under a laminar flow hood without delay. Prepare stoppers with a suitable antiseptic solution. Do not use unless solution is clear and seal is intact. Parenteral drug products should be inspected visually for particulate matter and discoloration prior to administration, whenever the solution and container permit. Ascorbic acid should be diluted in sterile water for injection (final volume of 1000ml).

The ascorbic acid or placebo for ascorbic acid infusions will be prepared by the pharmacy. The calculated dose of ascorbic acid will be diluted in Sterile Water for Injection USP. Normal saline will be used for the placebo infusion. To maintain the blind for the study, the infusion bags will be covered with opaque bags and tubing will be covered with an opaque tubing cover or amber tape.

A study-specific worksheet will be completed when preparing ascorbic acid infusions. The worksheet will document the dose prepared and calculations for dose preparation.

8.1.5 Storage

Store between 2°- 8°C. Do not freeze.

8.1.6 Stability

The administration of ascorbic acid infusion must be completed within 72 hours of preparation. If not used immediately, the infusion solution must be refrigerated while also protected from light to keep the product stable. The maximum 72 hour period under refrigeration and protected light conditions includes the product administration period.

8.1.7 Route of Administration

Ascorbic acid should be administered by intravenous infusion. For intravenous injection, dilution into a large volume parenteral of water is recommended to minimize the adverse reactions associated with intravenous injection. Infusion will be administered at 0.75-1 gram per minute not to exceed 500 mL per hour. Ports are recommended but not mandatory due to frequency of infusion.

Treatment will be administered on an outpatient basis with the option of home infusions starting week 2 of the cycle. If home infusions are administered, then institutional guidelines must be followed. Docetaxel infusions and week 1 of every cycle must be administered on an outpatient basis with the exception of the week 1 day 4 ascorbic acid/placebo infusion, which can be given at home when clinically appropriate. Reported

adverse events and potential risks are described in Section 7. Appropriate dose modifications are described in Section 6. No investigational or commercial agents or therapies other than those described below may be administered with the intent to treat the patient's malignancy. At randomization, the assignment of ascorbic acid versus placebo will be blinded to the patients and investigators.

8.1.8 Patient Care Implications

Too rapid intravenous administration of the solution may cause temporary faintness or dizziness.

Diabetics, patients prone to recurrent renal calculi, those undergoing stool occult blood tests, and those on sodium-restricted diets or warfarin anticoagulant therapy[18,19] should not take excessive doses of ascorbic acid over an extended period of time. Diabetics taking more than 500 mg of ascorbic acid daily may obtain false readings of their urinary glucose test. No exogenous ascorbic acid should be ingested for 48 to 72 hours before amine dependent stool occult blood tests are conducted because possible false-negative results may occur. Heparin, enoxaparin, aspirin, NSAIDS, or other anticoagulant therapies are allowed.

Ascorbic acid at pharmacologic doses can give false positive results for blood glucose with some commercial point of care glucometers (glucose meters), although this has not seen with clinical chemistry laboratory autoanalyzers. Glucometers should not be used to measure blood glucose during and up to 10 hours after intravenous ascorbate administration[20,21]. Patients should be counseled that correction of falsely high blood glucose levels with excess insulin may cause hypoglycemia and death, that some strips provide false readings while others, such as Bayer Contour are less likely to do so. They should also be counseled that if they do see high blood glucose readings following IV ascorbic acid, they should not increase their insulin unless the high glucose reading is confirmed at least 10 hours after IV AA administration.

8.1.9 Returns and Reconciliation

The investigator is responsible for keeping accurate records of the clinical supplies, the amount dispensed to and returned by the patients, and the amount remaining at the conclusion of the trial.

Upon completion or termination of the study, all unused and/or partially used investigational products will be destroyed at the site per institutional policy. It is the Investigator's responsibility to arrange for disposal of all empty containers, provided that procedures for proper disposal have been established according to applicable federal, state, local, and institutional guidelines and procedures, and provided that appropriate records of disposal are kept.

8.2 Docetaxel (Taxotere)

8.2.1 Agent Accountability

The sponsor/investigator shall take responsibility for and shall take all steps to maintain appropriate records and ensure appropriate supply, storage, handling, distribution, and

usage of investigational product in accordance with the protocol and any applicable laws and regulations. Docetaxel is commercially supplied.

8.2.2 Mode of Action

Docetaxel is an antineoplastic agent (belonging to the taxoid family) that acts by disrupting the microtubular network in cells that is essential for mitotic and interphase cellular functions. Docetaxel binds to free tubulin and promotes the assembly of tubulin into stable microtubules while simultaneously inhibiting their disassembly. This leads to the production of microtubule bundles without normal function and to the stabilization of microtubules, which results in the inhibition of mitosis in cells. Docetaxel's binding to microtubules does not alter the number of protofilaments in the bound microtubules, a feature which differs from most spindle poisons currently in clinical use.

8.2.3 Description

Docetaxel Injection Concentrate is a clear yellow to brownish-yellow viscous solution. Docetaxel is sterile, non-pyrogenic, and is available in single-dose vials containing 20 mg (1 mL) or 80 mg (4 mL) Docetaxel (anhydrous). Each mL contains 20 mg Docetaxel (anhydrous) in 0.54 grams polysorbate 80 and 0.395 grams dehydrated alcohol solution.

The Injection Concentrate is supplied in a single-dose vial as a sterile, pyrogen-free, nonaqueous, viscous solution with an accompanying sterile, nonpyrogenic, diluent (13% ethanol in Water for Injection) vial.

8.2.4 Preparation

Docetaxel should be reconstituted according to the manufacturer's recommendation.

8.2.5 Storage

Docetaxel should be stored between 2 and 25°C (36-77°F) in a secure and dry place. Retain in the original package to protect from bright light. Freezing does not adversely affect the product.

8.2.6 Stability

Taxotere infusion solution, if stored between 2 and 25°C (35.6 and 77°F) is stable for 4 hours. Fully prepared Taxotere infusion solution (in either 0.9% Sodium Chloride solution or 5% Dextrose solution) should be used within 4 hours (including the administration).

8.2.7 Route of Administration

Docetaxel should be administered by intravenous infusion.

8.2.8 Patient Care Implications

The most common adverse reactions across all TAXOTERE indications are infections, neutropenia, anemia, febrile neutropenia, hypersensitivity, thrombocytopenia, neuropathy, dysgeusia, dyspnea, constipation, anorexia, nail disorders, fluid retention, asthenia, pain, nausea, diarrhea, vomiting, mucositis, alopecia, skin reactions, and myalgia. Incidence varies depending on the indication.

Drug interactions: Cytochrome P450 3A4 inducers, inhibitors, or substrates: May alter docetaxel metabolism.

8.2.9 Returns and Reconciliation

Not applicable.

9. CORRELATIVE/SPECIAL STUDIES

9.1 Plasma Samples (Optional)

In patients willing to undergo an optional PK collection, we will determine whether ascorbic acid alters docetaxel exposure and compare between treatment arms, pharmacokinetics samples will be collected prior to, during, and after docetaxel infusions on Cycle 1 Day 1 (docetaxel in combination with ascorbic acid administration). PK samples should be collected from a separate site from where the drug is being infused, such as a contralateral arm or either arm if using a port. If there are severe adverse events that are greater than what is expected by the treating physician and thought to be related to study drug, PKsare strongly recommended to help determine etiology of AEs. NOTE: If PKs are collected for AEs, the collection will start on the next dose in the subsequent cycle.

9.1.1 Docetaxel Pharmacokinetics Samples (Optional)

Blood samples will be collected for docetaxel pharmacokinetics on Cycle 1 Day 1 (docetaxel after ascorbic acid administration) at the following time points:

PK for patients consenting to the optional PK.
Day 1 of Cycle 1:
Prior to docetaxel administration
Document the start of infusion time
30 minutes after the start of the docetaxel infusion
55 minutes after the start of the docetaxel infusion and prior to end of infusion
Document the end of infusion time
2 hours post- end of infusion docetaxel infusion (~3 hours)
5 hours post- end of infusion docetaxel infusion (~6 hours)

Day 2 of Cycle 1:
Approximately 24 hours post-docetaxel infusion

PK for patients who had more frequent or severe toxicities.
Day 1 of the next Cycle (Cycle >2):
Prior to docetaxel administration
Document the start of infusion time
30 minutes after the start of the docetaxel infusion
55 minutes after the start of the docetaxel infusion and prior to end of infusion
Document the end of infusion time
2 hours post- end of infusion docetaxel infusion (~3 hours)
5 hours post- end of infusion docetaxel infusion (~6 hours)

Day 2 of the next Cycle (Cycle >2):
Approximately 24 hours post-docetaxel infusion

Docetaxel pharmacokinetics samples will be measured using a validated liquid chromatography/tandem mass spectrometric (LC/MS/MS) method in the Sidney Kimmel Comprehensive Cancer Center Analytical Pharmacology Core (APC) Laboratory. If clinically significant differences in efficacy or toxicity are noted between treatment arms, then unbound docetaxel exposure and alpha-1-acid glycoprotein will be assessed for a comprehensive assessment of docetaxel pharmacology. Unbound docetaxel will be determined using equilibrium dialysis methods using a radiolabeled docetaxel or LC/MS/MS. Alpha-1-acid glycoprotein will be determined utilizing a Radial Immunodiffusion Plates from Kent Diagnostics.

Relevant individual PK parameters will be estimated using a compartmental PK method with the software WinNonlin. The pharmacokinetic variables will be tabulated and descriptive statistics (e.g., geometric means and coefficients of variation) calculated for each treatment arm. If clinically significant differences in efficacy or toxicity are noted between treatment arms, the unbound concentrations and alpha-1-acid glycoprotein will be incorporated into a population PK method with NONMEM or PKBugs to ascertain the differences between arms. Pharmacokinetic parameters (i.e., C_{max}, AUC $T_{½}$, and Cl) will be compared across treatment using nonparametric statistical testing techniques. Correlations with toxicity and efficacy will be explored using nonparametric statistical testing techniques. Significance for comparisons will be at the $p<0.05$ level.

Refer to the study lab manual for processing and shipping instructions.

9.1.2 Ascorbic Acid Pharmacokinetics Samples (Optional)

PK for patients consenting to the optional PK.
Blood samples will be collected for ascorbic acid pharmacokinetics on Cycles 1, 2, 3, 4, and 6 at the following time points:

First ascorbate administration of the cycle of Cycles 1, 2, 3, 4, and 6:
pre-ascorbic acid infusion
immediately after the end of the ascorbic acid infusion
60 minutes after the completion of the ascorbic acid infusion

Day 19 (or same day as last ascorbate administration of the cycle) of Cycles 1, 2, 3, 4, and 6:
pre-ascorbic acid infusion
immediately after the end of the ascorbic acid infusion
60 minutes after the completion of the ascorbic acid infusion

PK for patients who had more frequent or severe toxicities.
Blood samples will be collected for ascorbic acid pharmacokinetics on the next Cycle (Cycle >2) after the adverse drug event occurs at the following time points:

First ascorbate administration of the next cycle:
pre-ascorbic acid infusion
immediately after the end of the ascorbic acid infusion

60 minutes after the completion of the ascorbic acid infusion

Day 19 (or same day as last ascorbate administration of the next cycle):
pre-ascorbic acid infusion
immediately after the end of the ascorbic acid infusion
60 minutes after the completion of the ascorbic acid infusion

Ascorbic acid in plasma and in red blood cells will be measured by HPLC coupled to coulometric electrochemical detection using a validated method.

Refer to the study lab manual for processing and shipping instructions.

9.1.4 F_2-isoprostanes

Blood samples will be collected for F_2-isoprostanes at baseline (any time prior to first dose of docetaxel administration) and on Cycles 4 and 6 at the following time points:

Baseline (pre-treatment) sample:
Any time prior to first dose of docetaxel treatment

First ascorbate administration of the cycle of Cycles 4 and 6:
pre-ascorbic acid infusion
immediately after the end of the ascorbic acid infusion
60 minutes after the completion of the ascorbic acid infusion

Correlative analyses will assess the association between ascorbic acid and lipid peroxidation (F_2-isoprostanes) in the two study arms, globally, and over time. F_2-isoprostanes are a reliable marker of oxidative stress recommended for assessing oxidant injuries in humans. We hypothesize that higher levels of ascorbic acid in the blood will be associated with reduced toxicity and that the mechanism of action is through the reduction of F_2-isoprostanes. For each patient, we will divide each follow-up laboratory data value of ascorbic acid and F_2-isoprostanes by its baseline value and perform a log transformation if necessary to symmetrize the data. Initially these data will be summarized graphically by study arm and time point using scatterplots and boxplots with data points superimposed.

Two types of analyses of the correlates will be done. In the first, repeated measurements of toxicity and the correlates will be averaged over time points and analyses done at the patient level. Analysis of covariance models (ANCOVA) will be used to relate toxicity with ascorbic acid and F_2-isoprostanes, allowing for differences in the two treatment arms.

The second type of analysis will be based on the repeated measures data. Comparisons of ascorbic acid and F_2-isoprostanes by study arm at Cycle 4 and Cycle 6, accounting for baseline measures obtained from the same patient, will be made by taking differences between post baseline and baseline values and comparing these differences between arms of the study with t-tests. This comparison of differences may be adjusted for the presence of toxicity using a regression model. Regression will also be used to assess the association between F_2-isoprostane and ascorbic acid at Cycle 4 and Cycle 6, adjusting for study arm and toxicity.

Plasma F_2-Isoprostanes will be measured by a validated LC/MS method.

Refer to the study lab manual for processing and shipping instructions.

9.2 Serum Samples

Blood will be collected at Day 1 of each cycle (must be pre-treatment on Cycle 1 Day 1), and at study completion (30-day follow-up visit) to identify potential therapeutic targets, biomarkers, and predictors of response through proteomic approaches. Refer to the study lab manual for processing and shipping instructions.

10. STUDY CALENDAR

**Required Studies**	**Pre-study**	**R**	**Cycles 1-8 (1 cycle = 21 days)**							**30-day FU[17]**	**Long FU[7]**
			**Day 1**	**Day 2**	**Day 4**	**Day 8**	**Day 12**	**Day 15**	**Day 19**		
Visit Windows (days)[1]	-21 to -7	-7 to 0								± 7	± 28
**TREATMENT**											
Docetaxel			X								
Ascorbic acid/Placebo[2,3]			X		X	X	X	X	X		
**ELIGIBILITY**											
Inclusion/exclusion review	X										
Informed consent	X										
Randomization		X									
**PHYSICAL**											
Medical and Cancer History	X										
Physical Exam and ECOG [4]	X		X[4]							X	
Vital Signs, Weight[5]	X		X[5]			X		X		X	
AE/Toxicity Notation			X[4]		X	X	X	X	X	X	
Concurrent meds	X		X[4]			X		X		X	
FACT-P Questionnaire[6]	X		X[6]								
Long term follow-up[7]											X
**LABORATORY**											
Chemistry[8,9,10]	X		X			X		X		X	
Hematology[8,9,10]	X		X							X	
PSA[9]	X		X							X	
Urinalysis	X										
G6PD[11]	X										
**CORRELATIVE[18]**											
Docetaxel PK Samples[12]			X[12]	X[12]							
Ascorbic Acid PK Samples[13]			X[13]						X[13]		
Plasma samples for F_2-isoprostanes[14]	X[14]		X[14]								
Serum Samples[15]			X							X	
**DISEASE ASSESSMENT**											
Radiologic Evaluation/ RECIST 1.1[1, 16] or PCWG2	X[1]		X[16]							X[16]	

1 Longer delays to be approved by the study sponsor. IRB approval is required for all eligibility waivers prior to randomization. The window for the

baseline Radiologic Evaluation/RECIST 1.1 is 4 weeks.

[2] Each treatment cycle is 3 weeks (21 days). Ascorbic Acid/Placebo dosing should be 2 times per week on day 1 of each cycle and any day as long as it is at least 24 hours from the prior infusion.

[3] Ports are recommended but not mandatory due to the frequency of infusion.

[4] After screening, physical exams may be limited as indicated by symptoms. ECOG – Eastern Cooperative Oncology Group performance status. Physical exam, ECOG status, AE/Toxicity notation and concurrent medication review have a -4 day window of day 1 of the cycle.

[5] Vital signs include temperature, blood pressure, respiration rate, heart rate, and weight. Height is collected pre-study only study. There is a -4 day window of day 1 of the cycle that the vital signs can be collected within.

[6] To assess changes in quality of life measures, the FACT-P questionnaire will be given at baseline (Pre-Study or Cycle 1 Day 1), Cycle 4 Day 1, Cycle 6 Day 1; and Cycle 8 Day 1. There is a -4 day window of day 1 of the cycle that the FACT-P questionnaire can be completed within.

[7] Survival status and subsequent therapies will be collected during long term follow-up (LTFU) every 6 months (+/- 4 weeks), by phone or chart review, for 3 years or until study closure. Gather date of death, date of radiographic and/or PSA progression, start and stop dates for subsequent therapies.

[8] Clinical Hematology: CBC with differential ANC, ALC, AEC, and platelet count; Serum Chemistry: sodium, potassium, chloride, bicarbonate, glucose, BUN, creatinine, ALT, AST, alkaline phosphatase, total bilirubin, total protein, albumin, calcium.

[9] Safety labs may be collected within a window of up to five days prior to scheduled visit/dosing.

[10] Labs should be reassessed approximately once per week for hold parameters. Only labs required for ascorbic hold parameters will be required on weeks 2 and 3. A CBC does not need to be drawn unless clinically indicated and would not alter dosing with placebo/ascorbic acid.

[11] G6PD testing: Red blood cell hemolysis may occur in people found to be deficient in the G6PD enzyme.

[12] PK is optional. If there are severe adverse events that are greater than what is expected by the treating physician and thought to be related to study drug, PKs are strongly recommended to help determine etiology of AEs. For docetaxel PK samples, blood should be drawn during Day 1 of Cycle 1 at the following time points: prior to docetaxel administration, 30 and 55 minutes after the start of the docetaxel infusion, and 2 and 5 hours post-docetaxel infusion; Day 2: approximately 24 hours post-docetaxel infusion. See protocol Section 9.1.1 for details.

[13] PK is optional. If there are severe adverse events that are greater than what is expected by the treating physician and thought to be related to study drug, PKs are strongly recommended to help determine etiology of AEs. For ascorbic acid correlative analysis, approximately 5 mL of whole blood will be collected for plasma and RBC isolation at each time point. Blood should be drawn during Cycles 1, 2, 3, 4, and 6 at the following time points: first ascorbate administration of the cycle: Pre-ascorbic acid infusion, immediately after the ascorbic acid infusion, 60 minutes after the completion of the ascorbic acid infusion, and last ascorbate administration of the cycle: pre-ascorbic acid infusion, immediately after the ascorbic acid infusion, and 60 minutes after the completion of the ascorbic acid infusion. In addition when feasible, samples will be requested from the patient in the event of DLT or other persistent and severe toxicity during any cycle of therapy. See protocol Section 9.1.2 and 9.1.3 for details.

[14] For F_2-isoprostanes correlative samples, approximately 10 mL of whole blood will be collected for plasma isolation at each time point. Blood should be drawn at baseline (1 sample, any time prior to first dose of docetaxel administration) and on the first ascorbate administration of the cycle of Cycles 4 and 6 at the following time points: Pre-ascorbic acid infusion, immediately after the ascorbic acid infusion, and 60 minutes after the completion of the ascorbic acid infusion. See protocol Section 9.1.4 for details.

[15] Approximately 10mL of whole blood will be collected for serum isolation at each time point. See protocol Section 9.2 for details.

[16] CT scan (chest/abdomen/pelvis or MRI if patient has contrast allergy) and bone scan to be assessed at baseline and every 12 weeks (+/- 1 week), and at the 30-day follow-up visit (scans do not need to be repeated if one has not been done within the past 6 weeks).

[17] 30 days after their last dose of study drug. Ascorbic acid will be available twice a week until progression. Patients will be required to get safety labs (CBC and CMP weekly for ascorbic acid), and be assessed for progression per investigator judgment and at least every 12 weeks by PSA, CMP, CBC, bone scan, CT scan, and H&P.

[18] Research samples will be collected at the discretion of the PI based on availability of supplies and safety of patient and staff.

NOTE: *In order to minimize the need for research-only in-person visits, telemedicine visits may be substituted for in-person clinical trial visits or portions of clinical trial visits where determined to be appropriate and where determined by the investigator not to increase the participants risks. Prior to initiating telemedicine for study visits the study team will explain to the participant, what a telemedicine visit entails and confirm that the study participant is in agreement and able to proceed with this method. Telemedicine acknowledgement will be obtained in accordance with the Guidance for Use of Telemedicine in Research. In the event telemedicine is not deemed feasible, the study visit will proceed as an in-person visit. Telemedicine visits will be conducted using HIPAA compliant method approved by the Health System and within licensing restrictions.*

11. DATA REPORTING / REGULATORY REQUIREMENTS

Adverse event lists, guidelines, and instructions for AE reporting can be found in Section 7.0 (Adverse Events: List and Reporting Requirements).

11.1 Data Management

All information will be collected on study-specific case report forms (CRFs) by study staff. These data will be reviewed for completeness and accuracy by the Principal Investigator.

Data and/or completed case report forms must be transmitted by fax or e-mail to the Coordinating Center following the completion of each cycle as detailed in Section 10. Case report forms will be provided to participating sites by the Coordinating Center.

11.2 Safety Meetings

Scheduled meetings will take place weekly, biweekly or monthly depending on patient enrollement, and will include the protocol principal investigator, study coordinator(s), data manager(s), sub-investigators (as appropriate), collaborators (as appropriate), and biostatisticians (as appropriate) involved with the conduct of the protocol. During these meetings matters related to the following will be discussed: safety of protocol participants, validity and integrity of the data, enrollment rate relative to expectation, characteristics of participants, retention of participants, adherence to protocol (potential or real protocol violations), data completeness, and progress of data for objectives.

11.3 Monitoring

The SKCCC Compliance Monitoring Program will provide external monitoring for JHU-affiliated sites in accordance with SKCCC DSMP (Version 6.0, 02/21/2019). The SMC Subcommittee will determine the level of patient safety risk and level/frequency of monitoring.

Dr. Channing Paller will be holding the IND for this study. She will comply with all regulated reporting requirements to the FDA.

Authorized representatives of the Coordinating Center may visit participating sites to perform audits or inspections, including source data verification. The purpose of these audits or inspections is to systematically and independently examine all trial related activities and documents to determine whether these activities were conducted and data were recorded, analyzed, and accurately reported according to the protocol, Good Clinical Practice (GCP), and any applicable regulatory requirements.

Interim analysis of toxicity, outcome and ongoing scientific investigations will be performed every 6 months by the Sidney Kimmel Comprehensive Cancer Center Data Safety Monitoring Board (SKCCC DSMB). The SKCCC DSMB will review aspects of this trial that are outlined in the responsibilities section of the Data and Safety Monitoring Board (DSMB) Guidance. If the committee decides that amendments should be made to

this trial, recommendations will be made in writing to the Study Principal Investigator. The study team will submit modifications to the IRB within 60 days of receipt from the DSMB. The Associate Director of Clinical Research, will arbitrate any disagreements between the DSMB and the study Principal Investigator. These changes may include early termination of accrual if deemed appropriate.

12. STATISTICAL CONSIDERATIONS

12.1 Study Design

The hypotheses of this study are that I.V. ascorbic acid may increase PSA responses over the 24 weeks of treatment and/or mitigate toxicities over this same time period. This study will have toxicity and PSA response as co-primary endpoints. PSA response will be defined as achieving at least a 50% decline in PSA from baseline (day 1, prior to treatment initiation) at any time during the 24 weeks of treatment. Toxicity will be measured as the worst grade of four specific adverse events (AEs) over the 24 weeks of treatment. The specific toxicities for this endpoint are fatigue, nausea, bone pain, and anorexia. Data from the TAX 327 study, which enrolled similar patients, show a PSA response rate of 45% and an overall grade 3-4 AE rate of 45.8% (all toxicity types). It is expected for the current trial that docetaxel with I.V. ascorbic acid will improve the PSA response rate to 80%. While we do not have preliminary data for the toxicity endpoint as defined for this study, we include simulation results that illustrate the probability the study will detect a significant difference between the two treatment arms. Scenarios for these simulations are based on the TAX 327 study.

Primary objective: Determine if the proportion of patients with ≥50% decline in PSA over the 8 cycles of therapy is higher in the docetaxel with I.V. ascorbic acid arm and determine if the proportions of AEs of fatigue, nausea, bone pain, and anorexia reported in the docetaxel+ I.V. ascorbic acid arm are lower compared to the docetaxel and placebo arm.

Co-Primary endpoints:

1. The occurrence of a ≥50% decline in PSA from baseline at any time during the 24 weeks of treatment.
2. The proportions of worst grade AEs among those specified (fatigue, nausea, bone pain, and anorexia) in the categories: no toxicities, grade 1-2, and grade 3-4 in the two arms of the study.

Secondary objectives:

1. Compare radiographic progression free survival (rPFS).
2. Compare the proportions of maximum grade serious adverse events (SAE) of fatigue, nausea, bone pain, and anorexia during the 24 weeks of treatment.
3. Compare the proportions of maximum grade SAE of all types.
4. Compare QoL as measured by the FACT-P questionnaire.
5. Compare docetaxel dose reductions.
6. Compare overall survival between arms of the study.

Correlative objectives:

1. To determine whether ascorbic acid alters docetaxel exposure and compare between treatment arms. To correlate docetaxel exposure with clinical outcomes.
2. To determine peak and trough ascorbic acid levels
3. As a pharmacodynamic measure of oxidant injury in vivo, measure F_2-isoprostanes

12.2 Sample Size/Accrual Rate

PSA response and toxicity are co-primary endpoints of this study. To preserve the study significance level at 15%, the significance level is set at 5% for PSA response and 10% for toxicity. The TAX 327 PSA response rate used as the reference for this study is 45%. A one-sided 0.05 Fisher's exact test with the sample size 63, 21 patients on the docetaxel+placebo arm and 42 patients on the docetaxel with I.V. ascorbic acid arm, has 80% power to detect an improvement in the PSA response rate to 80%.

Sample size implications for the toxicity endpoint are summarized below. Power determination is based on simulated clinical studies in which the analysis is the Cochran-Armitage test for trend comparing two groups in observed proportions scored by toxicity grade (i.e., categorized as grade 0, grades 1-2, and grades 3-4). All scenarios assume that, on average, 16% of patients on the docetaxel+placebo arm experience a grade 3-4 toxicity of one of the specified types. This estimate was obtained as the sum of grade 3-4 AEs for fatigue, nausea, bone pain, and anorexia reported for the every-3-week docetaxel arm in the Tannock study[16].

Scenario 1: Substantial decreases in both high and medium grade toxicities:
After 5000 simulations at the 0.1-alpha level with probabilities

	Grade 0	Grade 1-2	Grade 3-4
P0 standard	0.10	0.74	0.16
P1 Vit C	0.45	0.50	0.05

Rejected 4654 times (93.1%)

Scenario 2: Smaller decreases in high and medium grade toxicities:
After 5000 simulations at the 0.1-alpha level with probabilities

	Grade 0	Grade 1-2	Grade 3-4
P0 standard	0.10	0.74	0.16
P1 Vit C	0.30	0.60	0.10

Rejected 2629 times (52.6%)

Scenario 3: No decrease in high grade toxicities, but large decrease in medium grade toxicity:
After 5000 simulations at the 0.1-alpha level with probabilities

	Grade 0	Grade 1-2	Grade 3-4
P0 standard	0.10	0.74	0.16
P1 Vit C	0.50	0.34	0.16

Rejected 3711 times (74.2%)

Scenario 4: No decrease in high grade toxicity, but some decrease in medium grade toxicity:
After 5000 simulations at the 0.1-alpha level with probabilities

	Grade 0	Grade 1-2	Grade 3-4

P0 standard	0.10	0.74	0.16
P1 Vit C	0.34	0.50	0.16

Rejected 1971 times (39.4%)

Scenario 5: No change in toxicity at any level:
After 5000 simulations at the 0.1-alpha level with probabilities

	Grade 0	Grade 1-2	Grade 3-4
P0 standard	0.10	0.74	0.16
P1 Vit C	0.10	0.74	0.16

Rejected 497 times (9.9%)

12.3 Monitoring Plan

We will monitor the PSA response endpoint for futility midway through the trial. The PSA endpoint will be monitored using a predictive probability approach. This monitoring plan is based on calculations of the predicted probability of a significant result if the trial were to continue to the end. At the time the trial is monitored, 500 simulations of the future results of the trial are generated and analyzed. If the probability is too low (i.e., less than 0.05) that the trial would end with a significant result, the study may be stopped for futility. The study will be monitored after 30 patients, 10 patients in the docetaxel+placebo arm and 20 patients in the docetaxel with I.V. ascorbic acid arm. The predictive distributions of the number of events in future patients are generated using a beta-binomial distribution with α and β parameters based on the data observed in the trial up to that point.

The table below gives the operating characteristics of the monitoring plan based on 1000 simulations for different combinations of p0 and p1. The first row shows results using the hypothesized proportions used for the study design (p0=0.45) and (p1=0.80). Other combinations for p0 and p1 are simulated to demonstrate the stopping rule if the difference in these proportions is smaller than hypothesized.

Table 1. Operating characteristics of monitoring rule for PSA response

p0 vs p1	Prob stop for futility	Avg N/group (Control, Trt)	Prob H0 rejected (power)
**0.45 vs 0.80**	**3.2%**	**20.6, 41.3**	**79.6%**
0.45 vs 0.76	5.9%	20.4, 40.7	66.8%
0.45 vs 0.70	12.2%	19.7, 39.3	51.6%
0.45 vs 0.60	27.4%	18.0, 36.0	22.4%
0.45 vs 0.45	53.9%	15.1, 30.1	3.3%

Safety monitoring and early stopping rule for safety:
A central hypothesis of this study is that I.V. ascorbate will help alleviate pain and reduce treatment related toxicities. Since we also do not anticipate overlapping toxicities of I.V. ascorbate and docetaxel, we will monitor the study arm for two types of toxicities that, if observed, would likely to be attributable to the addition of I.V. This includes sclerosis preventing infusion and kidney stones requiring cessation of treatment. Safety will be monitored after every patient in the

docetaxel+I.V, ascorbate study arm using 33% as the target limit on these adverse events. This monitoring rule will halt enrollment if the posterior probability of these events exceeding 0.33 is 70% or higher. The prior probability for this toxicity monitoring rule will be a Beta distribution with parameters of 2 and 4. This distribution corresponds to assuming a 2 in 5 chance that the risk of DLT is 33% or higher and 90% certainty that the risk is between 7.6% and 65.7%. The monitoring rule applies this prior distribution to the observed number of patients experiencing events and computes the resulting posterior probability that the rate is too high. If the posterior certainty that the rate is too high based on these assumptions is 70% or higher, the study should stop. The following table shows the resulting guideline.

Study paused if:	2 AEs	3 AEs	4 AEs	5 AEs	6 AEs	7 AEs
And patients between:	2-3	4-6	7-8	9-11	12-14	15-17

Study paused if:	8 AEs	9 AEs	10 AEs	11 AEs	12 AEs	13 AEs
And patients between:	18-20	21-22	23-25	26-28	29-31	32-34

Study paused if:	14 AEs	15 AEs	16 AEs
And patients between:	35-37	38-40	41-42

For example, the rule will call for stopping the study if 4 out of the first 7 patients experience these AEs. The next table shows the percent of the time that the stopping rule will terminate the study under different hypothetical risks of AEs, along with the average sample size (based on 5000 simulations).

Risk of AE	0.10	0.20	0.25	0.30	0.35	0.40	0.45	0.50
% of Time Study Stops	4.2%	19.5%	34.6%	50.9%	71.1%	86.3%	95.2%	98.7%
Expected Sample Size	40.4	35.0	30.2	25.5	19.4	14.1	10.1	7.5

12.4 Analysis Plans

Co-Primary endpoints:
The primary analysis for the PSA response outcome will be a one-sided 0.05 alpha level Fisher's exact test. The primary analysis for the toxicity endpoint will be a one-sided 0.10 Cochran-Armitage test for trend.

Secondary endpoints:

1. For each treatment arm, the median rPFS and a 2-sided 95% confidence interval will be provided. Estimates of the time-to-event curves from the Kaplan-Meier method will be provided and compared using the log-rank statistic.
2. The proportions of patients experiencing grade 3 or higher (maximum grade) serious adverse events (SAEs) of fatigue, nausea, bone pain, and/or anorexia during the 24 weeks of treatment will be compared between arms of the study with the Cochran-Armitage test for trend.
3. Proportions of maximum grade SAE of all types will also be compared using the Cochran-

Armitage test for trend.

4. The FACT-P questionnaire will be given at baseline and once prior to cycles 4, 6 and 8 of therapy. FACT-P scores will be compared at each cycle between arms of the study with an analysis of covariance using the baseline QoL score as a covariate.
5. The number of dose reductions and total number of completed cycles will be summarized by study arm.
6. For each treatment arm, the median OS and a 2-sided 95% confidence interval will be provided. Estimates of the time-to-event curves from the Kaplan-Meier method will be provided and compared using the log-rank statistic.

Correlative Analyses:
Correlative analyses will assess the association between ascorbic acid and lipid peroxidation (F_2-isoprostanes) in the two study arms, globally, and over time. F_2-isoprostanes are a reliable marker of oxidative stress recommended for assessing oxidant injuries in humans. We hypothesize that higher levels of ascorbic acid in the blood will be associated with reduced toxicity and that the mechanism of action may be coupled to reductions of F_2-isoprostanes. For each patient, we will divide each follow-up laboratory data value of ascorbic acid and F_2-isoprostanes by its baseline value and perform a log transformation if necessary to symmetrize the data. Initially these data will be summarized graphically by study arm and time point using scatterplots and boxplots with data points superimposed.

Two types of analyses of the correlates will be done. In the first, repeated measurements of toxicity and the correlates will be averaged over time points and analyses done at the patient level. Analysis of covariance models (ANCOVA) will be used to relate toxicity with ascorbic acid and F_2-isoprostanes, allowing for differences in the two treatment arms.

The second type of analysis will be based on the repeated measures data. Comparisons of ascorbic acid and F_2-isoprostanes by study arm at cycle 4 and cycle 6, accounting for baseline measures obtained from the same patient, will be made by taking differences between post baseline and baseline values and comparing these differences between arms of the study with t-tests. This comparison of differences may be adjusted for the presence of toxicity using a regression model. Regression will also be used to assess the association between F_2-isoprostane and ascorbic acid at cycle 4 and at cycle 6, adjusting for study arm and toxicity.

Relevant individual PK parameters will be estimated using a compartmental PK method with the software WinNonlin. The pharmacokinetic variables will be tabulated and descriptive statistics (e.g., geometric means and coefficients of variation) calculated for each treatment arm. If clinically significant differences in efficacy or toxicity are noted between treatment arms, the unbound concentrations and alpha-1-acid glycoprotein will be incorporated into a population PK method with NONMEM or PKBugs to ascertain the differences between arms. Pharmacokinetic parameters (i.e., C_{max}, AUC $T_{½}$, and Cl) will be compared across treatment using nonparametric statistical testing techniques. Correlations with toxicity and efficacy will be explored using nonparametric statistical testing techniques. Significance for comparisons will be at the $p<0.05$ level.

12.5 Expected accrual rate, accrual duration, and total study duration

We plan to open the study at four institutions: Johns Hopkins Hospital/Sibley Memorial Hospital, Karmanos, Jefferson, University Hospitals of Cleveland Seidman Cancer Center and Inova. The accrual rate is expected to be one to two patients per month at each institution, with the completion of accrual expected within 18 months. Total accrual at Johns Hopkins/Sibley is expected to be 25 patients, with 13 from each of the other institutions.

REFERENCES

1 Dagher, R. *et al.* Approval summary: Docetaxel in combination with prednisone for the treatment of androgen-independent hormone-refractory prostate cancer. *Clinical cancer research : an official journal of the American Association for Cancer Research* **10**, 8147-8151, doi:10.1158/1078-0432.CCR-04-1402 (2004).

2 Hoffer, L. J. *et al.* Phase I clinical trial of i.v. ascorbic acid in advanced malignancy. *Annals of oncology : official journal of the European Society for Medical Oncology / ESMO* **19**, 1969-1974, doi:10.1093/annonc/mdn377 (2008).

3 Monti, D. A. *et al.* Phase I evaluation of intravenous ascorbic acid in combination with gemcitabine and erlotinib in patients with metastatic pancreatic cancer. *PloS one* **7**, e29794, doi:10.1371/journal.pone.0029794 (2012).

4 Welsh, J. L. *et al.* Pharmacological ascorbate with gemcitabine for the control of metastatic and node-positive pancreatic cancer (PACMAN): results from a phase I clinical trial. *Cancer chemotherapy and pharmacology* **71**, 765-775, doi:10.1007/s00280-013-2070-8 (2013).

5 Stephenson, C. M., Levin, R. D., Spector, T. & Lis, C. G. Phase I clinical trial to evaluate the safety, tolerability, and pharmacokinetics of high-dose intravenous ascorbic acid in patients with advanced cancer. *Cancer chemotherapy and pharmacology* **72**, 139-146, doi:10.1007/s00280-013-2179-9 (2013).

6 Ma, Y. *et al.* High-dose parenteral ascorbate enhanced chemosensitivity of ovarian cancer and reduced toxicity of chemotherapy. *Science translational medicine* **6**, 222ra218, doi:10.1126/scitranslmed.3007154 (2014).

7 Vollbracht, C. *et al.* Intravenous vitamin C administration improves quality of life in breast cancer patients during chemo-/radiotherapy and aftercare: results of a retrospective, multicentre, epidemiological cohort study in Germany. *In vivo* **25**, 983-990 (2011).

8 Padayatty, S. J. *et al.* Vitamin C: intravenous use by complementary and alternative medicine practitioners and adverse effects. *PloS one* **5**, e11414, doi:10.1371/journal.pone.0011414 (2010).

9 Chen, Q. *et al.* Pharmacologic ascorbic acid concentrations selectively kill cancer cells: action as a pro-drug to deliver hydrogen peroxide to tissues. *Proceedings of the National Academy of Sciences of the United States of America* **102**, 13604-13609, doi:10.1073/pnas.0506390102 (2005).

10 Chen, P. *et al.* Pharmacological ascorbate induces cytotoxicity in prostate cancer cells through ATP depletion and induction of autophagy. *Anti-cancer drugs* **23**, 437-444, doi:10.1097/CAD.0b013e32834fd01f (2012).

11 Pollard, H. B., Levine, M. A., Eidelman, O. & Pollard, M. Pharmacological ascorbic acid suppresses syngeneic tumor growth and metastases in hormone-refractory prostate cancer. *In vivo* **24**, 249-255 (2010).

12 Parrow, N. L., Leshin, J. A. & Levine, M. Parenteral ascorbate as a cancer therapeutic: a reassessment based on pharmacokinetics. *Antioxidants & redox signaling* **19**, 2141-2156, doi:10.1089/ars.2013.5372 (2013).

13 Levine, M., Padayatty, S. J. & Espey, M. G. Vitamin C: a concentration-function approach yields pharmacology and therapeutic discoveries. *Advances in nutrition* **2**, 78-88, doi:10.3945/an.110.000109 (2011).

14 de Weger, V. A., Beijnen, J. H. & Schellens, J. H. Cellular and clinical pharmacology of the taxanes docetaxel and paclitaxel--a review. *Anti-cancer drugs* **25**, 488-494, doi:10.1097/CAD.0000000000000093 (2014).

15 Puisset, F. *et al.* Clinical pharmacodynamic factors in docetaxel toxicity. *British journal of cancer* **97**, 290-296, doi:10.1038/sj.bjc.6603872 (2007).

16 Tannock, I. F. *et al.* Docetaxel plus prednisone or mitoxantrone plus prednisone for advanced prostate cancer. *The New England journal of medicine* **351**, 1502-1512, doi:10.1056/NEJMoa040720 (2004).

17 Scher, H. I. *et al.* Design and end points of clinical trials for patients with progressive prostate cancer and castrate levels of testosterone: recommendations of the Prostate Cancer Clinical Trials Working Group. *Journal of clinical oncology : official journal of the American Society of Clinical Oncology* **26**, 1148-1159, doi:10.1200/JCO.2007.12.4487 (2008).

18 Feetam, C. L., Leach, R. H. & Meynell, M. J. Lack of a clinically important interaction between warfarin and ascorbic acid. *Toxicology and applied pharmacology* **31**, 544-547 (1975).

19 Sattar, A., Willman, J. E. & Kolluri, R. Possible warfarin resistance due to interaction with ascorbic acid: case report and literature review. *American journal of health-system pharmacy : AJHP : official journal of the American Society of Health-System Pharmacists* **70**, 782-786, doi:10.2146/ajhp110704 (2013).

20 Dimeski, G., Jones, B. W., Tilley, V., Greenslade, M. N. & Russell, A. W. Glucose meters: evaluation of the new formulation measuring strips from Roche (Accu-Chek) and Abbott (MediSense). *Annals of clinical biochemistry* **47**, 358-365, doi:10.1258/acb.2010.009291 (2010).

21 Vasudevan, S. & Hirsch, I. B. Interference of intravenous vitamin C with blood glucose testing. *Diabetes care* **37**, e93-94, doi:10.2337/dc13-2452 (2014).

22 Berenson, J.R., Matous, J., Swift R.A., Mapes, R., Morrison, B. & Yeh, H.S., *et a*l. A phase I/II study of arsenic trioxide/bortezomib/ascorbic acid combination therapy for the treatment of relapsed or refractory multiple myeloma. DOI:10.1158/1078-0432.CCR-06-1812

APPENDIX A: Performance Status Criteria

ECOG Performance Status Scale		Karnofsky Performance Scale	
Grade	Descriptions	Percent	Description
0	Normal activity. Fully active, able to carry on all pre-disease performance without restriction.	100	Normal, no complaints, no evidence of disease.
		90	Able to carry on normal activity; minor signs or symptoms of disease.
1	Symptoms, but ambulatory. Restricted in physically strenuous activity, but ambulatory and able to carry out work of a light or sedentary nature (e.g., light housework, office work).	80	Normal activity with effort; some signs or symptoms of disease.
		70	Cares for self, unable to carry on normal activity or to do active work.
2	In bed <50% of the time. Ambulatory and capable of all self-care, but unable to carry out any work activities. Up and about more than 50% of waking hours.	60	Requires occasional assistance, but is able to care for most of his/her needs.
		50	Requires considerable assistance and frequent medical care.
3	In bed >50% of the time. Capable of only limited self-care, confined to bed or chair more than 50% of waking hours.	40	Disabled, requires special care and assistance.
		30	Severely disabled, hospitalization indicated. Death not imminent.
4	100% bedridden. Completely disabled. Cannot carry on any self-care. Totally confined to bed or chair.	20	Very sick, hospitalization indicated. Death not imminent.
		10	Moribund, fatal processes progressing rapidly.
5	Dead.	0	Dead.

APPENDIX B: FACT-P Questionnaire (Version 4)

APPENDIX C: Serious Adverse Event Reporting Form

Please notify IND Sponsor, Channing Paller, MD within 24 hours (Fax: 410-614-8397, Email: cpaller1@jhmi.edu)

<table>
<tr><td>Protocol Title:</td><td colspan="3">A Randomized Phase 2 Trial of Ascorbic Acid in Combination with Docetaxel in Men with Metastatic Prostate Cancer</td></tr>
<tr><td>Protocol Number:
J15106</td><td>Signature of PI:</td><td>Principal Investigator:</td><td>Date:</td></tr>
<tr><td>Report Type:
☐Initial
☐Follow-up
☐Final Follow-up
☐Death
☐Addendum to:</td><td>Serious Criteria (check all that apply):
☐Death
☐Life-threatening
☐ Hospitalization or Elongation of Existing Hospitalization
☐ Persistent or Significant Disability
☐ Congenital Anomaly
☐ Other Important Medical Event
☐ Cancer
☐ Overdose</td><td>Hospital Admission Date:

Hospital Discharge Date:</td><td>Date Event Discovered:</td></tr>
</table>

<table>
<tr><td colspan="3">Section A: Subject Information</td></tr>
<tr><td>Subject ID:</td><td>Subject Initial:</td><td>Subject Gender:
☐Male
☐Female</td></tr>
</table>

<table>
<tr><td colspan="3">Section B: Event Information</td></tr>
<tr><td rowspan="3">Event diagnosis or symptoms:</td><td>Date of First Dose:</td><td rowspan="3">Action taken with the study drug:

☐None
☐Interrupted
☐Discontinued
☐Delayed</td></tr>
<tr><td>Date of Last Dose prior to Event:</td></tr>
<tr><td>Number of Total Doses:</td></tr>
</table>

**Event Onset Date:**	**Event End Date:**

Relationship to:	Ascorbic acid/Placebo	Docetaxel	Underlying Disease
Unrelated	☐	☐	☐
Related	☐	☐	☐

**Section C: Brief Description of the Event:**

**Section D: Relevant Medical History**

Section E: Concomitant Drug (Not related to Serious Adverse Event)

Name of the Drug	Start Date	Stop Date	Route	Dose	Frequency

**Section F: Comments**
**Additional Documents:** ☐ Please specify
